# A TBK1 variant causes autophagolysosomal and motoneuron pathology without neuroinflammation in mice

**DOI:** 10.1084/jem.20221190

**Published:** 2024-03-22

**Authors:** David Brenner, Kirsten Sieverding, Jahnavi Srinidhi, Susanne Zellner, Christopher Secker, Rüstem Yilmaz, Julia Dyckow, Shady Amr, Anna Ponomarenko, Esra Tunaboylu, Yasmin Douahem, Joana S. Schlag, Lucía Rodríguez Martínez, Georg Kislinger, Cornelia Niemann, Karsten Nalbach, Wolfgang P. Ruf, Jonathan Uhl, Johanna Hollenbeck, Lucas Schirmer, Alberto Catanese, Christian S. Lobsiger, Karin M. Danzer, Deniz Yilmazer-Hanke, Christian Münch, Philipp Koch, Axel Freischmidt, Martina Fetting, Christian Behrends, Rosanna Parlato, Jochen H. Weishaupt

**Affiliations:** 1 Division of Neurodegeneration, Department of Neurology, Medical Faculty Mannheim, Mannheim Center for Translational Neurosciences, Heidelberg University, Mannheim, Germany; 2 Department of Neurology, University of Ulm, Ulm, Germany; 3 Medical Faculty, Munich Cluster for Systems Neurology (SyNergy), Ludwig-Maximilians-University München, Munich, Germany; 4 Neuroproteomics, Max Delbrück Center for Molecular Medicine, Berlin, Germany; 5 Department of Neurology and Experimental Neurology, Charité-Universitätsmedizin Berlin, Corporate Member of Freie Universität Berlin and Humboldt-Universität zu Berlin, Berlin, Germany; 6 Division of Neuroimmunology, Department of Neurology, Medical Faculty Mannheim, Mannheim Center for Translational Neurosciences, Heidelberg University, Mannheim, Germany; 7 Faculty of Medicine, Institute of Biochemistry II, Goethe University Frankfurt, Frankfurt, Germany; 8 Institute of Anatomy and Cell Biology, Ulm University School of Medicine, Ulm, Germany; 9 Institute of Neuronal Cell Biology, Technical University Munich, Munich, Germany; 10 German Center for Neurodegenerative Diseases, Munich, Germany; 11 Electron Microscopy Hub, German Center for Neurodegenerative Diseases, Munich, Germany; 12 Institut du Cerveau—Paris Brain Institute—Institut du Cerveau et de la Moelle épinière, Inserm, Centre National de la Recherche Scientifique, Assistance Publique–Hôpitaux de Paris, Hôpital de la Pitié-Salpêtrière, Sorbonne Université, Paris, France; 13 German Center for Neurodegenerative Diseases, Ulm, Germany; 14 Department of Neurology, Clinical Neuroanatomy Unit, University of Ulm, Ulm, Germany; 15 University of Heidelberg/Medical Faculty Mannheim, Central Institute of Mental Health, Mannheim, Germany; 16 Hector Institute for Translational Brain Research, Mannheim, Germany; 17 German Cancer Research Center, Heidelberg, Germany

## Abstract

Heterozygous mutations in the TBK1 gene can cause amyotrophic lateral sclerosis (ALS) and frontotemporal dementia (FTD). The majority of TBK1-ALS/FTD patients carry deleterious loss-of-expression mutations, and it is still unclear which TBK1 function leads to neurodegeneration. We investigated the impact of the pathogenic *TBK1* missense variant p.E696K, which does not abolish protein expression, but leads to a selective loss of TBK1 binding to the autophagy adaptor protein and TBK1 substrate optineurin. Using organelle-specific proteomics, we found that in a knock-in mouse model and human iPSC–derived motor neurons, the p.E696K mutation causes presymptomatic onset of autophagolysosomal dysfunction in neurons precipitating the accumulation of damaged lysosomes. This is followed by a progressive, age-dependent motor neuron disease. Contrary to the phenotype of mice with full *Tbk1* knock-out, RIPK/TNF-α–dependent hepatic, neuronal necroptosis, and overt autoinflammation were not detected. Our in vivo results indicate autophagolysosomal dysfunction as a trigger for neurodegeneration and a promising therapeutic target in TBK1-ALS/FTD.

## Teaser

The ALS/FTD-associated TBK1 variant p.E696K shows a selective loss of optineurin binding. Its knock-in causes autophagolysosomal dysfunction and ALS-like symptoms in mice, while RIPK-dependent necroptosis or inflammation is absent. This highlights the role of autophagolysosomal dysfunction in TBK1-ALS/FTD.

## Graphical Abstract

**Figure:**
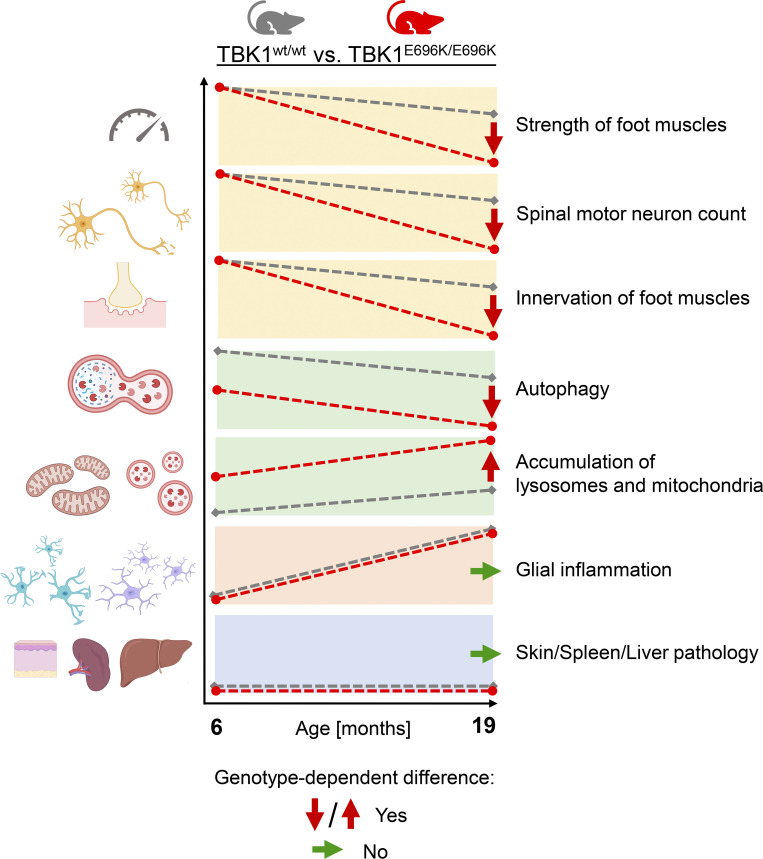

## Introduction

TANK-binding kinase 1 (TBK1) is a ubiquitously expressed Ser/Thr kinase. It regulates selective autophagy and innate immunity, in particular the type I IFN response, and inhibits RIPK1 (Ahmad et al., 2016; Oakes et al., 2017; Lafont et al., 2018). Mutations in *TBK1* cause amyotrophic lateral sclerosis (ALS) and frontotemporal dementia (FTD) (Freischmidt et al., 2015; Cirulli et al., 2015). Since almost all *TBK1*-linked ALS/FTD cases are caused by heterozygous deleterious mutations and thus the loss of one *TBK1* allele, the mechanism underlying *TBK1* mutations is most likely haploinsufficiency.

Motor neuron selective knock-out of *Tbk1* or a global heterozygous deletion alone does not cause motor neuron degeneration in aged mice (Gerbino et al., 2020; Bruno et al., 2020). Deficiency of *TBK1* as a result of homozygous loss-of-function mutations in the germline (i.e., in all cells) causes TNF-α– and RIPK1-dependent liver necrosis during embryonic development in mice and adult systemic autoinflammation in mice and humans (Marchlik et al., 2010; Xu et al., 2018; Taft et al., 2021). However, the relevance of these observations for neurodegeneration remains unclear since ALS mutations in *TBK1* are always heterozygous. Furthermore, the combination of a ubiquitous heterozygous deletion of *Tbk1* and a heterozygous myeloid cell–specific deficiency of *Tak1*, another inhibitor of RIPK1, has been reported to lead to cortical neurodegeneration and mild microglial neuroinflammation in RIPK1-dependent manner in mice (Xu et al., 2018). Again, it remains to be shown how the myeloid *Tak1* deletion in this model can be integrated into the overall view of ALS/FTD causation. Nevertheless, a clinical trial studying the effect of a RIPK1 inhibitor in ALS is ongoing (http://ClinicalTrials.gov identifier: NCT05237284).

Additional heterozygous knock-out of *Tbk1* impairs selective autophagy in spinal motor neurons of SOD1^G93A^ transgenic mice (Brenner et al., 2019; Gerbino et al., 2020). This finding points to a neuron-autonomous role of TBK1 at the beginning of the disease. Nevertheless, the overall consensus regarding the role of TBK1 and disease mechanisms of TBK1-linked ALS/FTD remained controversial. An important reason for this is that the above-mentioned animal models rely on a full deletion of the functionally pleiotropic TBK1 kinase. In contrast, the p.E696K variant is one of the few TBK1 missense variants with expression at the protein level and preserved kinase activity but proven ALS/FTD pathogenicity. It selectively abolishes the binding of the TBK1 protein to the autophagy adaptor protein optineurin (Freischmidt et al., 2015; Pottier et al., 2015; Richter et al., 2016; Li et al., 2016; Moore and Holzbaur, 2016). Therefore, the functional deficits caused by the p.E696K variant may not be representative for all *TBK1* mutations. However, the p.E696K variant represents a “minimally invasive” change that nevertheless is still sufficient to trigger ALS/FTD. Thus, it allows to more precisely define how TBK1 tunes ALS/FTD onset and progression. We thus took advantage of the p.E696K variant to generate the first *Tbk1* knock-in mouse model as well as induced pluripotent stem cell (iPSC)–derived human motor neurons carrying this mutation. In these models, we found evidence of early (autophago)lysosomal defects in spinal motor neurons, and *Tbk1*-linked neurodegeneration was not accompanied by overt neuroinflammation in the mouse model.

## Results and discussion

**Figure 1.**
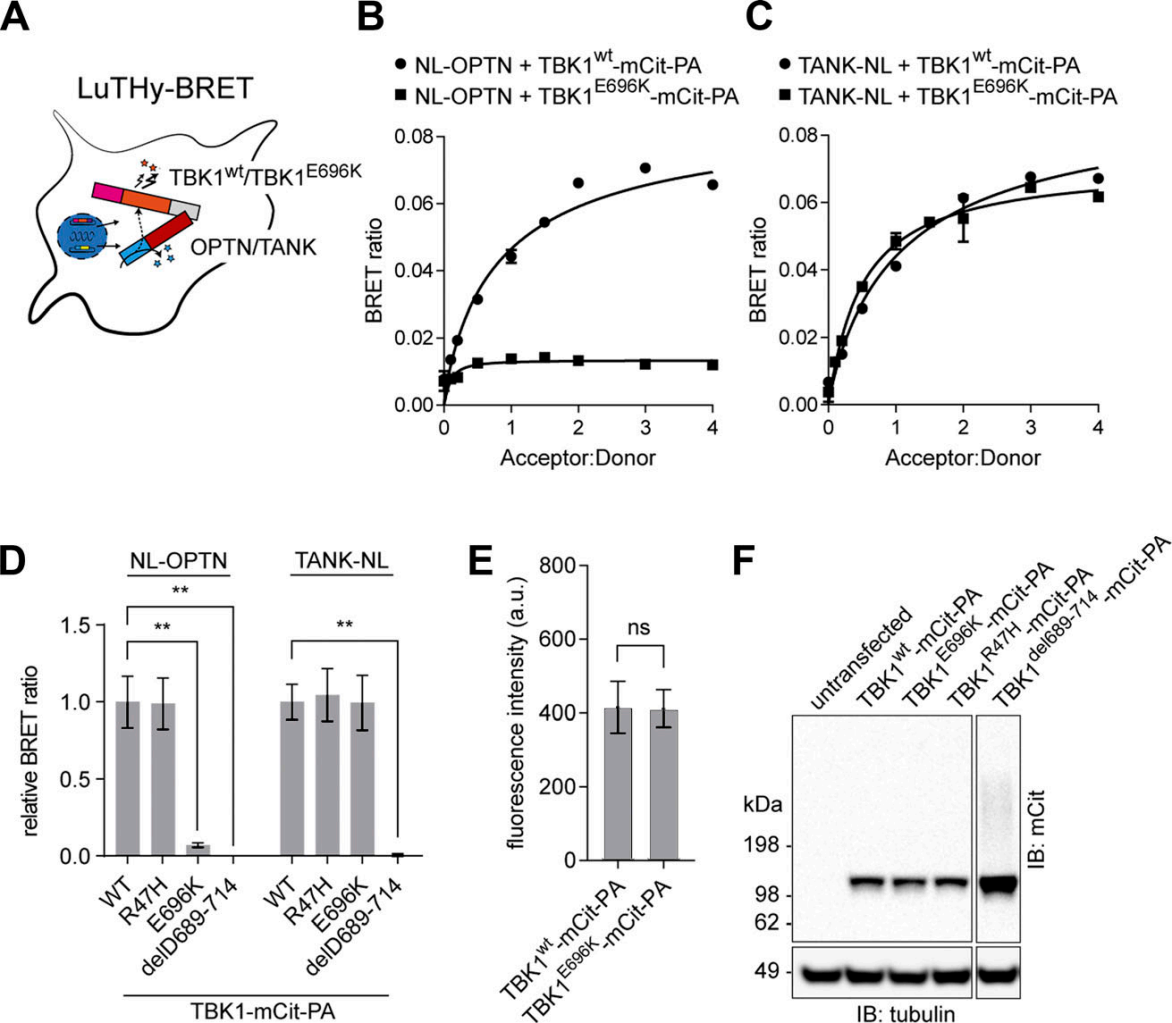
**Characterization of the TBK1**^**E696K**^ **variant. (A)** Scheme of LuTHy-BRET assay to investigate binding of wt TBK1 and TBK1^E696K^ to optineurin (OPTN) or the TRAF family member–associated NF-κB activator (TANK) in live HEK293 cells. **(B and C)** Binding of wt TBK1- and TBK1^E696K^-mCitrine-Protein A (-mCit-PA) to NanoLuc (NL)-tagged optineurin (B) or TANK (C) in LuTHy-BRET donor saturation assays. **(D)** Quantification of cBRET signals from TBK1 binding assays. PA-mCit-NL tandem construct shown as positive and cotransfection of single NL- and PA-mCit-tags as negative BRET controls. Relative BRET ratios were obtained by normalizing the BRET signal of optineurin or TANK with each TBK1 mutant to the interaction signals with wt TBK1, respectively. Mean ± SEM of *n* = 4 technical replicates per condition from three independent experiments; one-way ANOVA with Sidak’s post hoc test; **P < 0.01. **(E)** Comparison of wt TBK1- and TBK1^E696K^-mCit-PA fusion protein expression as measured from fluorescence intensities. Mean ± SEM of *n* = 7 technical replicates per condition from three independent experiments; Student’s *t* test. **(F)** Western blot showing expression of the TBK1 mutants in HEK293 cells after transfection. Source data are available for this figure: SourceData F1.

Almost all cases of *TBK1*-linked, dominantly inherited ALS and FTD are caused by loss-of-function mutations. However, not all pleiotropic functional deficits conferred by a full *TBK1*-KO may be necessary for ALS/FTD causation. Previous biochemical assays revealed that the p.E696K mutation selectively abolishes binding of TBK1 to the autophagy adaptor protein optineurin but retains binding to other autophagy adaptor proteins, its kinase activity and the ability to activate IFN transcription (Freischmidt et al., 2015; Pottier et al., 2015; Richter et al., 2016; Li et al., 2016; Moore and Holzbaur, 2016). Measuring the protein interactions of wildtype (wt) TBK1 (TBK1^wt^) and TBK1^E696K^ using the bioluminescence-based two-hybrid (LuTHy)–bioluminescence resonance energy transfer (BRET) assay (Trepte et al., 2018) (Fig. 1 A) confirmed the absence of TBK1^E696K^/optineurin interaction in HEK293 cells (Fig. 1, B and D), while TBK1^E696K^/TANK binding used as a positive control was preserved (Fig. 1, C and D). Deletion of a large part of the C-terminal protein binding domain in TBK1^del689-714^ led to the expected loss of optineurin and TANK binding (Fig. 1 D). Binding of TBK1 to optineurin and TANK was independent of TBK1 kinase activity since binding of the kinase domain mutant TBK1^R47H^ to both interaction partners was preserved (Fig. 1 D). Importantly, all TBK1 constructs showed similar expression levels (Fig. 1, E and F). Thus, we hypothesized that the TBK1^E696K^ mutation would allow to dissect the causative TBK1 pathogenic pathway in knock-in mice while avoiding the limitations of a complete loss of TBK1 function by a knock-out-based approach.

**Figure 2.**
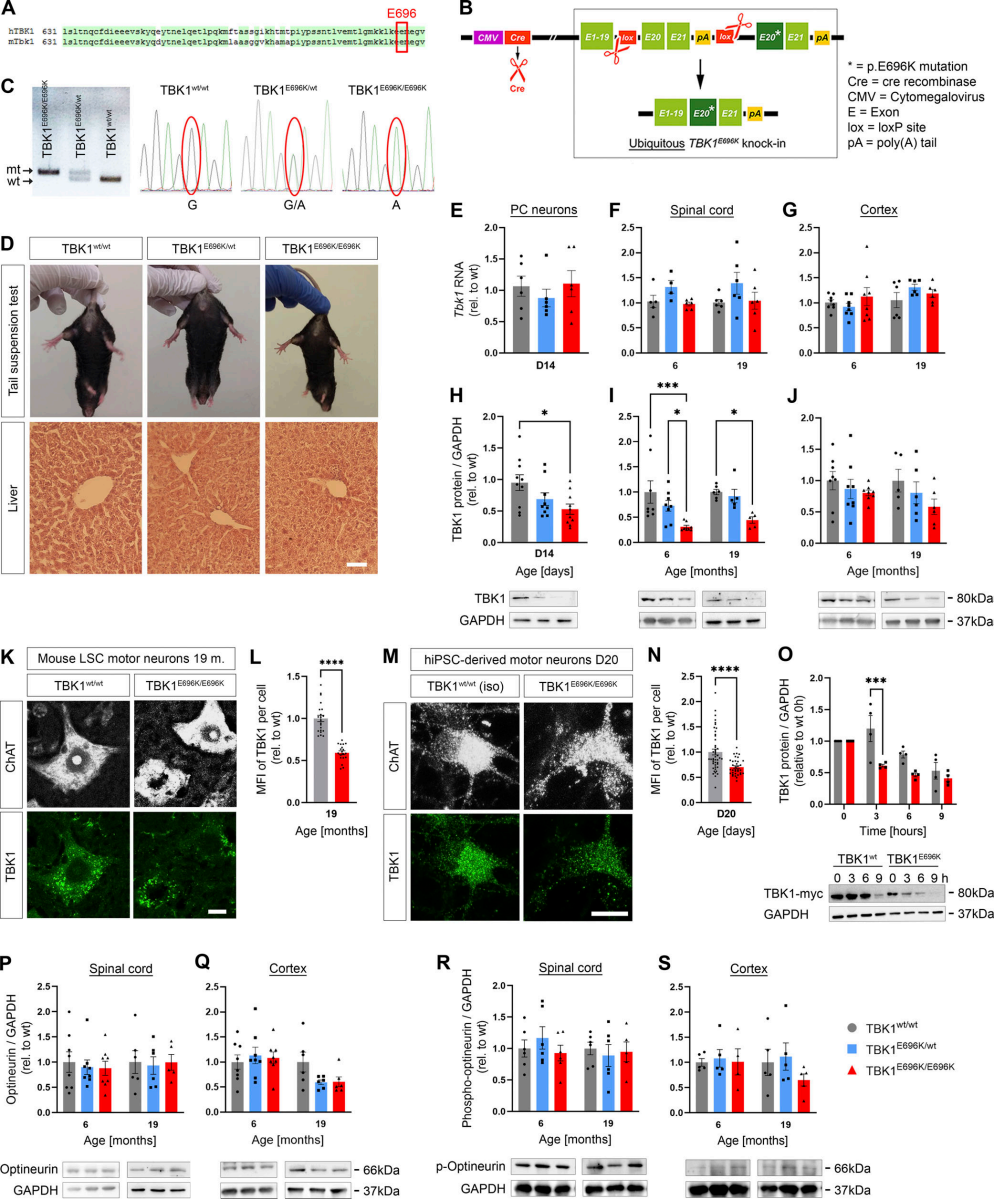
**Generation and characterization of TBK1**^**E696K**^ **knock-in mice. (A)** Conservation of p.E696 in TBK1 between mouse and human. **(B)** Scheme showing the generation of mice with constitutive global knock-in of the TBK1^E696K^ variant using the Cre/Lox system and a “mini-gene” approach. **(C)** PCR and sequencing of the wt and mutant bands of ear tissue from TBK1^E696K^ knock-in and wt siblings. **(D)** Representative photomicrographs of 19-mo-old male mice and light microscopic images of HE-stained livers show no morphological differences between the three genotypes. Scale bar: 100 µm. **(E–G)** Quantification of *Tbk1* RNA expression by qPCR in mouse primary cortical (PC) neurons, spinal cord, and cortex tissue. Mean ± SEM of *n* = 6 embryos of mixed sex per genotype form more than three independent experiments; one-way ANOVA with Tukey’s post hoc test in E. Mean ± SEM of *n* = 4–8 male mice per genotype; two-way ANOVA with Tukey’s post hoc test in F and G. **(H–J)** Western blot analysis of lysates of primary cortical neurons, spinal cord, and cortex tissue stained against TBK1 and GAPDH. Mean ± SEM of a pool of *n* = 9–10 embryos of mixed sex per genotype from more than three independent experiments; one-way ANOVA with Tukey’s post hoc test; *P < 0.05 in H. Mean ± SEM of *n* = 5–8 male mice per genotype from two independent experiments; two-way ANOVA with Tukey’s post hoc test in I and J; *P < 0.05; ***P < 0.001. The right GAPDH column from I is reused in Fig. S3, A and F. **(K)** Representative photomicrographs of LSC motor neurons of 17-mo-old TBK1^E696K^ knock-in and wt mice stained against Nissl and TBK1. Scale bar: 10 µm. **(L)** Quantification of the MFI of TBK1 fluorescence shows reduced expression in motor neurons of TBK1^E696K/E696K^ knock-in mice. Mean ± SEM of pool of *n* > 19 motor neurons from *n* = 5–6 male mice per genotype from two independent experiments; Student’s *t* test; ****P < 0.0001. **(M)** Representative photomicrographs of D20 hiPSC-derived motor neurons stained against ChAT and TBK1. Scale bar: 10 µm. **(N)** Quantification of MFI of TBK1 fluorescence shows reduced expression of TBK1 in TBK1^E696K/E696K^ mutant hiPSC-derived motor neurons. Mean ± SEM of pool of *n* > 35 motor neurons per genotype from two independent experiments; Mann–Whitney test; ****P < 0.0001. **(O)** Western blot analysis of HEK293 cells overexpressing myc-tagged wt TBK1 and TBK1^E696K^ after treatment with CHX. Mean ± SEM of *n* = 4 biological replicates from four independent experiments two-way ANOVA with post hoc Šídák’s multiple comparisons test; ***P < 0.001. **(P and Q)** Western blot analysis of lysates of spinal cord and cortex tissue stained against optineurin and GAPDH. Mean ± SEM of *n* = 6–8 male mice per genotype from two independent experiments; two-way ANOVA with Tukey’s post hoc test. **(R and S)** Western blot analysis of lysates of spinal cord and cortex tissue stained against phospho-optineurin and GAPDH. Mean ± SEM of *n* = 4–6 male mice per genotype from two independent experiments; two-way ANOVA with Tukey’s post hoc test. Source data are available for this figure: SourceData FS2.

Thus, we chose to generate a mouse strain with knock-in of the p.E696K mutation. This mutation resides in a domain of TBK1 that is 100% conserved between mice and humans at the protein level (Fig. 2 A). Since we planned to study also homozygous knock-in mice, we chose a conditional Cre-mediated knock-in strategy to by-pass the known TNF-α/RIPK1-dependent embryonic lethality and autoimmune infiltration of biallelic constitutive deleterious *Tbk1*/*TBK1* mutations in mice and humans (Marchlik et al., 2010; Xu et al., 2018) (Fig. 2 B and Materials and methods). Nevertheless, we could successfully generate mice with a constitutive homozygous TBK1^E696K^ germline knock-in (hereinafter called TBK1^E696K/E696K^) by breeding with mice expressing Cre under the ubiquitously active CMV promoter (see Materials and methods; Fig. 2 B). Surprisingly, the resulting constitutive TBK1^E696K/E696K^ mice (Fig. 2 C) were viable, born at the expected Mendelian ratio, and morphologically undistinguishable from wt mice (Fig. 2 D). We did not observe a genotype- or age-dependent difference in *Tbk1* RNA expression in primary cortical cells, spinal cord, or cerebral cortex lysates from TBK1^E696K/wt^, TBK1^E696K/E696K^, and wt mouse littermates (Fig. 2, E–G). However, analysis of protein levels by western blot revealed a significant reduction of the TBK1^E696K^ protein to about 50% of wt TBK1 in primary cortical neurons and CNS tissue from TBK1^E696K/E696K^ knock-in mice (Fig. 2, H–J). Quantification of the mean intensity of TBK1 immunofluorescence in spinal motor neurons from 19-mo-old mice and human iPSC (hiPSC)–derived motor neurons (see Materials and methods) confirmed the reduced levels of TBK1^E696K^ protein (Fig. 2 K–N) when compared with wt mice or isogenic control motor neurons, respectively. This is in line with reduced protein expression of TBK1^E696K^ in brain autopsy tissue from an ALS patient (Pottier et al., 2015). The TBK1^E696K^ protein did not show an altered distribution pattern compared with wt TBK1 in mice or motor neurons (Fig. 2, K and M). Cycloheximide (CHX) pulse chase analysis of protein degradation in HEK293 cells indicated a reduced stability of TBK1^E696K^ protein (Fig. 2 O). Optineurin and phospho-optineurin expression was not changed as assessed by western blotting of spinal cord and cerebral cortex protein lysates (Fig. 2, P–S).

**Figure S1.**
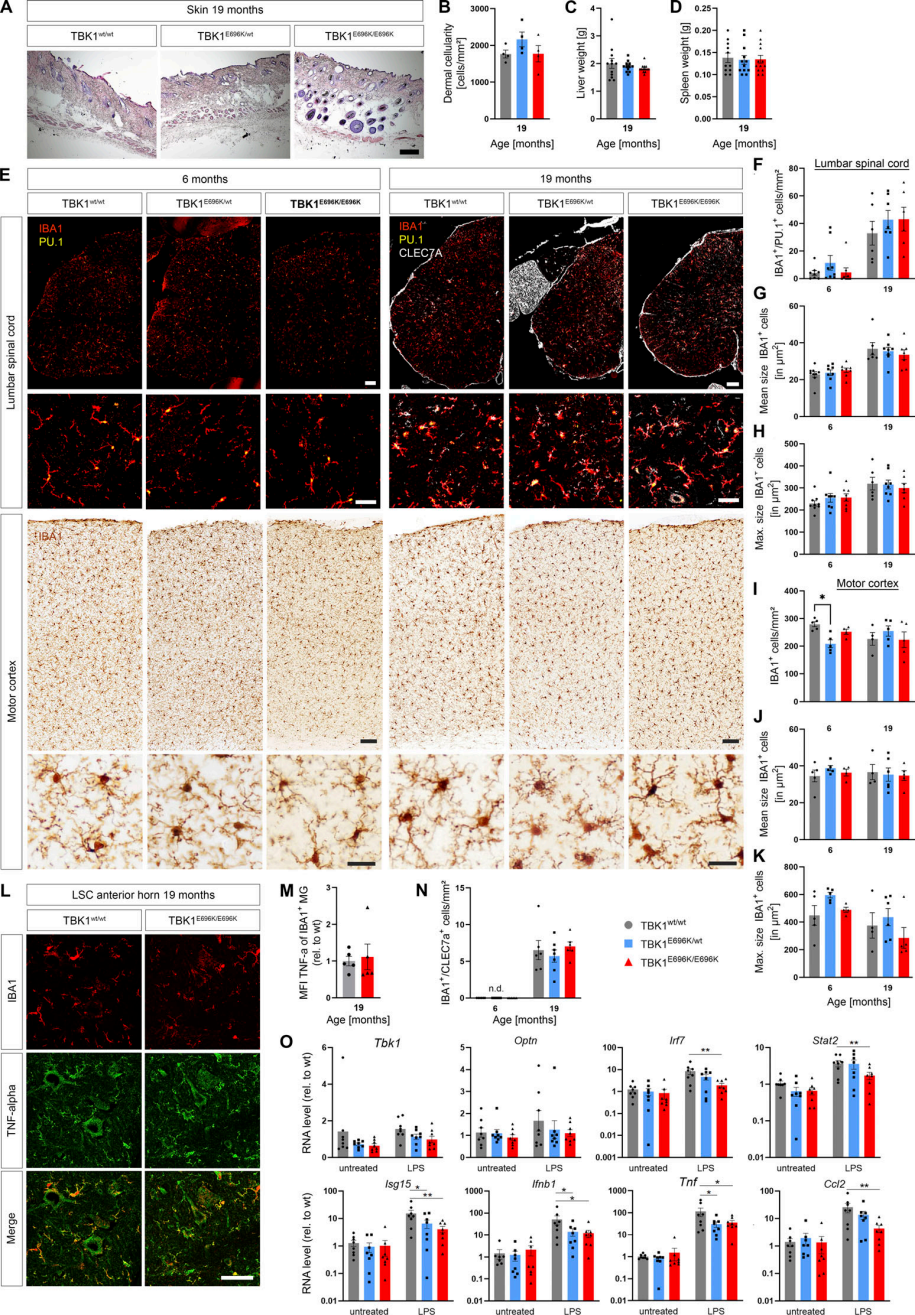
**Analysis of skin morphology, liver and spleen weight, and microglia count, morphology, and activation in the lumbar spinal cord and motor cortex from TBK1**^**E696K**^** knock-in and wt siblings. (A)** Representative light microscopic images of HE-stained skin tissue of 19-mo-old mice. Scale bar: 100 µm. **(B)** Quantification of dermal cellular density between 19-mo-old TBK1^E696K^ knock-in mice and wt siblings. Mean ± SEM of *n* = 4 male mice per genotype; one-way ANOVA with Tukey’s post hoc test. **(C and D)** Comparison of liver (C) and spleen (D) weights between 19-mo-old TBK1^E696K^ knock-in mice and wt siblings. Mean ± SEM of *n* = 13–15 male mice per genotype; one-way ANOVA with Tukey’s post hoc test. **(E)** Representative photomicrographs of LSC and motor cortex slices from 6- and 19-mo-old TBK1^E696K^ knock-in and wt mice stained against the microglial markers IBA1 and PU-1. Scale bars: 100 µm, 50 µm, 25 µm. **(F–K)** Analysis of abundances and mean and maximal sizes of microglia in LSC and motor cortex. Mean ± SEM of *n* = 6–8 male mice per genotype from two independent experiments; two-way ANOVA with post hoc Tukey’s multiple comparisons test; *P < 0.05. **(L)** Representative microscopic images of LSC anterior slices of 19-mo-old mice stained against IBA1 and TNF-α. Scale bar: 50 µm. **(M)** Quantification of the MFI of TNF-α in IBA1^+^ microglia. Mean ± SEM of *n* = 5 male mice per genotype from two independent experiments; Student’s *t* test. **(N)** Quantification of the abundance of IBA^+^/CLEC7A^+^ microglia from E. Mean ± SEM of *n* = 5–7 male mice per genotype from two independent experiments; one-way ANOVA with Tukey’s post hoc test. **(O)** qPCR of RNA transcripts of cultured primary microglia. Mean ± SEM of *n* = 8 pups of mixed sex from more than three independent experiments; two-way ANOVA with post hoc Tukey’s multiple comparisons test; *P < 0.05; **P < 0.01.

**Figure S2.**
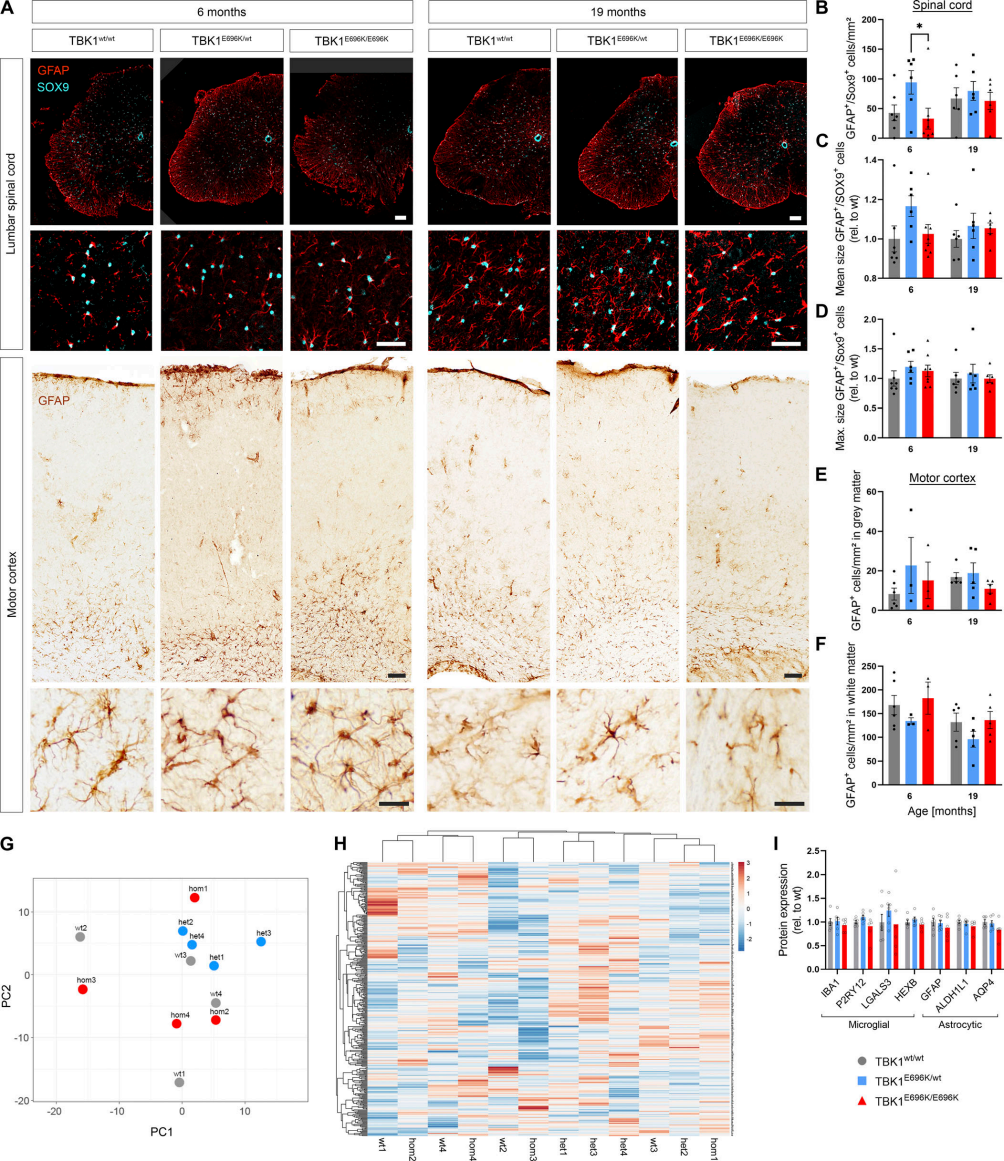
**Analysis of astrocyte count and morphology in the lumbar spinal cord and motor cortex as well as proteomic analysis of glial markers in the lumbar spinal cord from TBK1**^**E696K**^** knock-in and wt siblings. (A)** Representative photomicrographs of LSC and motor cortex slices from 6- and 19-mo-old TBK1^E696K^ knock-in and wt mice stained against the astrocytic markers GFAP and SOX9. Scale bars: 100 µm, 50 µm, 25 µm. **(B–F)** Quantification of the abundances, mean and maximal sizes of astrocytic cells in the spinal cord, and the gray and white matter of the motor cortex. Mean ± SEM of *n* = 3–8 male mice per genotype from two independent experiments; two-way ANOVA with post hoc Tukey’s multiple comparisons test; *P < 0.05 in B. **(G and H)** Principal component and heatmap hierarchical clustering analysis of glial proteins in LSC lysates from 19-mo-old mice does not show separation of the three genotypes. *N* = 4 male mice per genotype. **(I)** Expression of selected glial markers in LSC at 19 mo. Mean ± SEM of *n* = 6 male mice per genotype; two-way ANOVA with post hoc Tukey’s multiple comparisons test.

**Figure S3.**
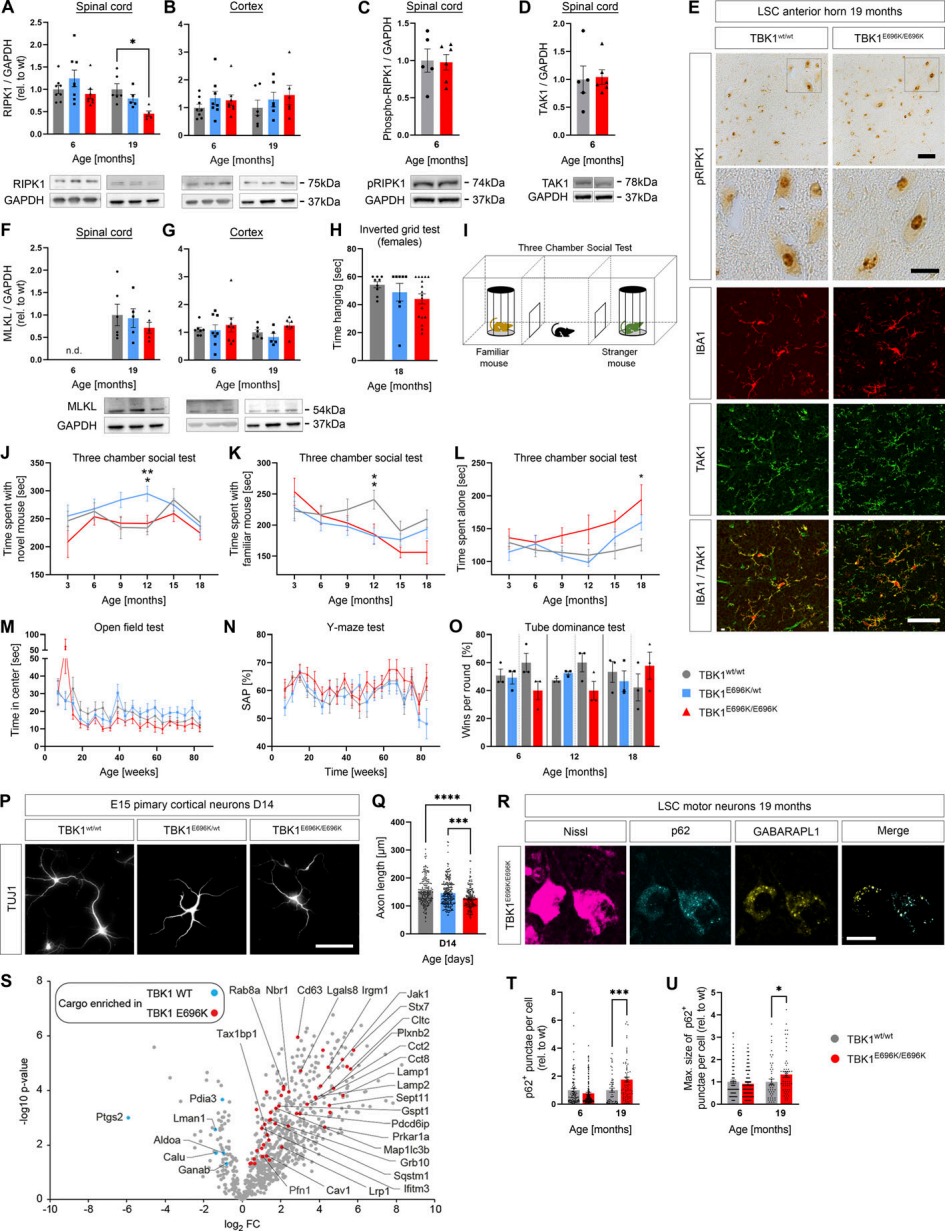
**Analysis of necroptosis, autophagy, and axon pathology as well as behavioral testing of TBK1**^**E696K**^** mutant and wt mice, MEFs, and primary neurons. (A and B)** Western blot analysis of LSC and cortex lysates from 6 to 19 mo old TBK1^E696K^ knock-in and wt mice stained against RIPK1. Mean ± SEM of *n* = 6 male mice per genotype from two independent experiments; two-way ANOVA with post hoc Tukey’s multiple comparisons test; *P < 0.05 in A. **(C and D)** Western blot analysis of LSC lysates from 6-mo-old TBK1^E696K^ knock-in and wt mice stained against pRIPK1 and TAK1. Mean ± SEM of *n* = 5–6 male mice per genotype from two independent experiments; Student’s *t* test. **(E)** Representative microscopic images of LSC anterior slices of 19-mo-old male mice stained against pRIPK1 (DAB) and TAK1 (IF). Scale bars: 50 µm, 25 µm (insets). **(F and G)** Western blot analysis of LSC and cortex lysates from 6- and 19-mo-old TBK1^E696K^ knock-in and wt mice stained against MLKL. Mean ± SEM of *n* = 6 male mice per genotype from two independent experiments; one-way ANOVA with Tukey’s multiple comparisons test in E; two-way ANOVA with post hoc Tukey’s multiple comparisons test in F. A (right column) and F use the same GAPDH blots used in Fig. 2 I (right column). B (left column) and G (left column) use the same GAPDH blots. B (right column) and G (right column) use the same GAPDH blots. **(H)** Inverted grid test in 18-mo-old female mice. Each time point represents mean ± SEM of *n* = 8–17 female mice per genotype; one-way ANOVA with Tukey’s post hoc test. **(I)** Scheme of three chamber social test. **(J–O)** Analysis of three chamber social test, open field test, Y-maze test, and tube dominance test. Each time point represents mean ± SEM of *n* = 13–15 male mice per genotype; mixed-effects analysis with post hoc Tukey’s multiple comparisons test; *P < 0.05; **P < 0.01. **(P)** Representative photomicrographs of hetero- and homozygous TBK1^E696K^ knock-in and wt E15 primary cortical neurons 14 days in culture and stained against TUJ-1. **(Q)** Homozygous TBK1^E696K^ knock-in primary cortical neurons show shortened axon lengths compared with the other genotypes. Mean ± SEM of pool of >160 motor neurons per genotype from *n* = 5–6 embryos of mixed sex from two independent experiments; Kruskal–Wallis test followed by Dunn’s multiple comparisons post hoc test; ***P < 0.001; ****P < 0.0001. **(R)** LSC section of a 19-mo-old TBK1^E696K^ knock-in mouse stained against Nissl, p62, and GABARAPL1 shows colocalization of both autophagy markers. Scale bar: 25 µm. **(S)** Volcano plot visualizing autophagosome content profiling in TBK1^E696K/E696K^ knock-in MEFs compared to wt. *N* = 4 technical replicates per condition from two independent experiments; multiple Student’s *t* tests without FDR correction; red/blue colors indicate significantly enriched proteins (uncorrected P < 0.05). **(T and U)** Analysis of abundance and maximal size of p62^+^ punctae in lumbar spinal motor neurons in 6- and 19-mo-old mice. Mean ± SEM of pool of *n* > 30 motor neurons from *n* = 4 male mice per genotype from two independent experiments; Mann–Whitney test; *P < 0.05; ***P < 0.001. Source data are available for this figure: SourceData FS3.

While mice with a complete knock-out of *Tbk1* suffered from embryonal liver necroptosis, splenomegaly, and systemic immune cell infiltration in the skin (Marchlik et al., 2010), this pathology was absent in TBK1^E696K/E696K^ knock-in mice (Fig. 2 D and Fig. S1, A–D). Dermal cellularity and weights of liver and spleen of TBK1^E696K/E696K^ knock-in animals were unaltered (Fig. S1, B–D). In addition, tissue immunostaining against the microglial markers Pu.1 and IBA1 did not show any changes in number, size, or morphology of microglia in TBK1^E696K^ knock-in mouse spinal cord and motor cortex (Fig. S1, E–K). Moreover, CLEC7A and TNF-α immunofluorescence in the spinal cord was comparable between wt and TBK1^E696K/E696K^ knock-in mice at the age of 19 mo (Fig. S1, E and L–N), while the expected age-dependent increase in activated microglia positive for CLEC7A was detected (Fig. S1 N). Cultured primary microglia with hetero- and homozygous TBK1^E696K^ knock-in even showed a reduced inflammatory gene expression profile including lower expression of IFN-I pathway members after stimulation with LPS compared with wt cells (Fig. S2 O).This observation is consistent with previous results from primary microglia with heterozygous deletion of *Tbk1* (Brenner et al., 2019). Because we observed a decreased expression of TBK1 in cortical neurons, tissues, and patient-derived motor neurons carrying the p.E696K mutation (Fig. 2, H–N), we propose that downregulation of IFN-I type cytokines is due to a reduced expression of TBK1^E696K^, and these effects become particularly manifest under conditions of strong stimulation, such as LPS. Further, staining against the astrocytic markers SOX9 and GFAP did not show any changes in the number and size or morphology of astrocytes in TBK1^E696K^ knock-in mouse spinal cord and motor cortex, further supporting the lack of an overt neuroinflammation phenotype (Fig. S2, A–F). To gain a broader view of the expression of glial proteins in the p.E696K knock-in mouse line, we performed a mass spectroscopy (MS) analysis of spinal cord lysates (Fig. S2, G–I; and Table S1). 360 out of 757 proteins from the nCounter Mouse Glial Profiling Panel that are expressed in glia cells could be detected in our proteomic data. Principal component analysis (PCA), hierarchical clustering, and heatmap analysis of these proteins did not distinguish between the hetero- or homozygous TBK1^E696K^ mutant mice from wt mice (Fig. S2, G and H; and Table S1). Unaltered abundance of seven established astrocytic and microglial marker proteins is shown in Fig. S2 I. Along these lines, protein levels of RIPK1, pRIPK1, its downstream effector MLKL as well as TAK1 were not altered in spinal cord and cortex lysates of TBK1^E696K/E696K^ knock-in mice, reinforcing the evidence that the p.E696K mutation has, if any, only minor implications on these proinflammatory pathways (Fig. S3, A–G). However, despite the lack of an overt neuroinflammatory phenotype, we cannot exclude that molecular analysis performed at a single-cell level by higher resolution tools, such as spatial transcriptomics, might reveal alterations of glial reactivity.

**Figure 3.**
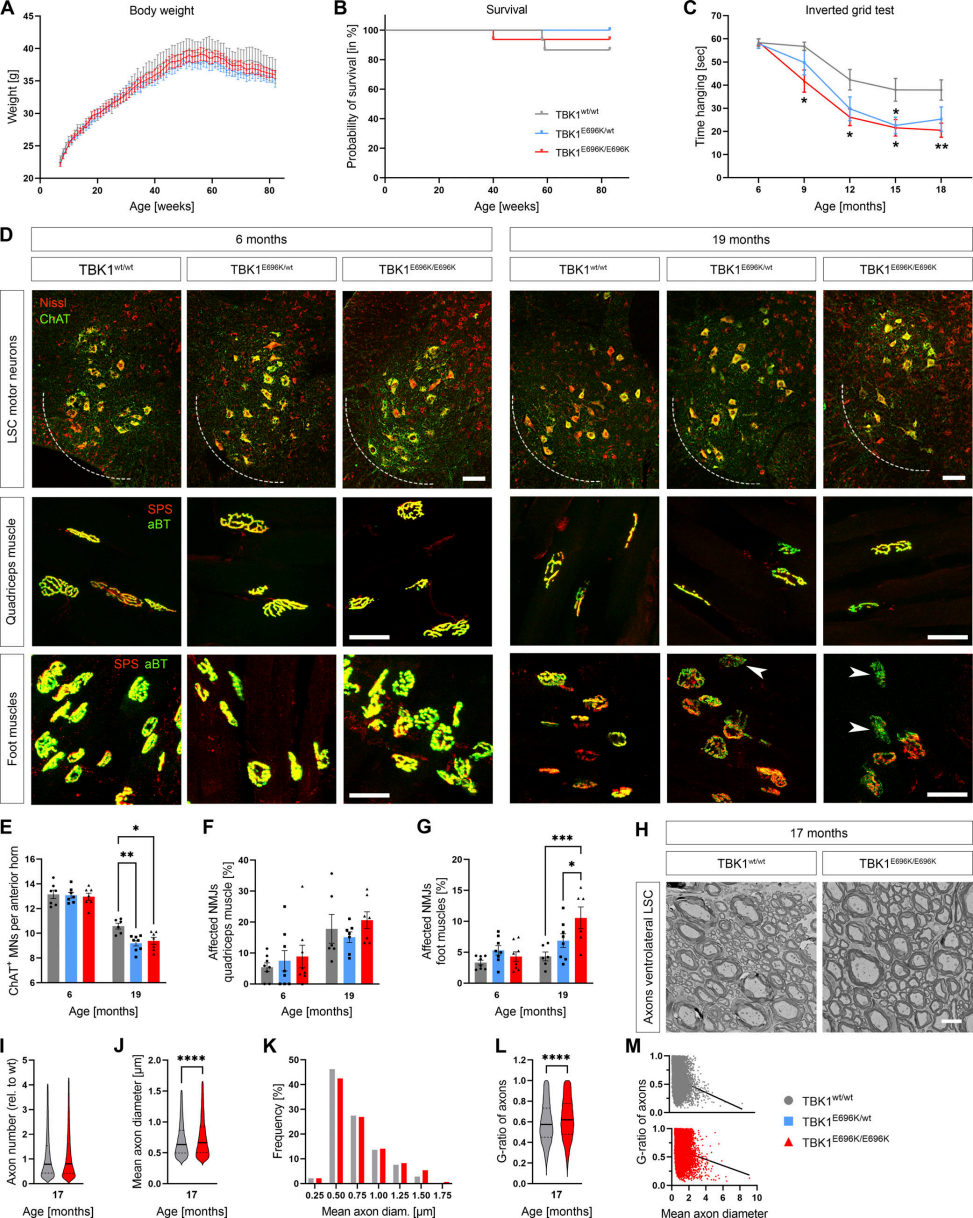
**Homozygous TBK1**^**E696K**^ **knock-in causes progressive motor neuron disease-like symptoms, muscle denervation, and spinal motor neuron loss in mice. (A)** TBK1^E696K^ knock-in and wt mice show similar weight kinetics. Each time point represents mean ± SEM of *n* = 13–15 male mice per genotype; mixed-effects analysis with post hoc Tukey’s multiple comparisons test. **(B)** Lifespan during the study period of 19 mo does not differ among the three genotypes. Each time point represents mean ± SEM of *n* = 13–15 male mice per genotype; log-rank (Mantel–Cox) test. **(C)** Compared to wt siblings, homozygous TBK1^E696K^ knock-in mice (TBK1^E696K/E696K^) show a progressively reduced latency to fall in the inverted grid test starting at the age of 9 mo. Each time point represents mean ± SEM of *n* = 13–15 male mice per genotype; mixed-effects analysis with post hoc Tukey’s multiple comparisons test; *P < 0.05; **P < 0.01. **(D)** Representative photomicrographs of proximal and distal muscles and lumbar anterior horns of 6- and 19-mo-old TBK1^E696K^ knock-in and wt mice stained against synaptophysin/α-bungarotoxin and ChAT/Nissl, respectively. Arrowhead indicates denervated NMJs. Scale bars: 100 µm (LSC) and 50 µm (muscles). **(E)** The anterior horn motor neuron count differs significantly between TBK1^E696K/E696K^ knock-in and wt mice at the age of 19 mo. Mean ± SEM of *n* = 6–8 male mice per genotype from four independent experiments; two-way ANOVA with post hoc Tukey’s multiple comparisons test; *P < 0.05; **P < 0.01. **(F and G)** Quantification of NMJ innervation reveals progressive denervation of the foot (distal) but not quadriceps (proximal) muscles in TBK1^E696K^ knock-in mice compared with wt siblings. Mean ± SEM of *n* = 6–8 male mice per genotype from two independent experiments; two-way ANOVA with post hoc Tukey’s multiple comparisons test; *P < 0.05; ***P < 0.001. **(H)** Representative TEM photomicrographs of axons in the ventrolateral LSC of 17-mo-old TBK1^E696K^ knock-in and wt mice. Scale bar: 5 µm. **(I–M)** Quantification of number, mean diameter (distribution), and g-ratio of axons shows a higher axon diameter and thinner myelin sheath in TBK1^E696K/E696K^ knock-in mice. Median ± quartiles of pool of *n* > 14,000 axons from *n* = 3 mice of mixed sex (2 males/1 female) per genotype from two independent experiments; Mann–Whitney test in each panel; ****P < 0.0001.

In conclusion, the ALS/FTD-causing TBK1^E696K^ mutation leads to a combined partial deficit of protein expression and complete loss of optineurin binding. There were no differences in body weight or lifespan between TBK1^E696K^ knock-in mice and wt controls (observation time up to 19 mo of age) (Fig. 3, A and B), in line with other single TBK1-mutant mouse strains (mice with motor neuron–selective homozygous knock-out of *Tbk1* and mice with global heterozygous deletion of *Tbk1*; Bruno et al., 2020; Gerbino et al., 2020; Sieverding et al., 2021). The absence of embryonic lethality, the preserved lifespan, the absence of liver necroptosis, and autoimmune disease or any signs of significant glial activation in the TBK1^E696K/E696K^ knock-in mice argue against a predominant role of necroptosis, neuroinflammation, and activation of the RIPK/TNF-α pathway for the p.E696K mutation-dependent, and possibly other TBK1-dependent ALS/FTD. These findings are consistent with our and other previous observations that heterozygous deletion of *Tbk1* reduced rather than increased *Ripk1* RNA levels in the spinal cord of 140-day-old SOD1^G93A^ mice (Brenner et al., 2019), and that constitutive deletion of *Ripk3* or *Mlkl*, which are both downstream effectors of RIPK1, in ALS SOD1^G93A^ mice did not improve behavioral or neuropathological deficits (Dermentzaki et al., 2019; Wang et al., 2020).

Despite the lack of overt neuroinflammation, however, the TBK1^E696K^ knock-in mutation induced a motor neuron disease phenotype in male mice. We observed progressive inverted grid test deficits in TBK1^E696K/E696K^ knock-in mice (strength of paw and foot muscles) from 9 mo onward (Fig. 3 C). Interestingly, heterozygous knock-in mice (TBK1^E696K/wt^) exhibited an intermediate inverted grid test performance between wt and homozygous knock-in mice (Fig. 3 C). A smaller group of female mice from surplus animal breeding was tested at the age of 18 mo only and revealed an inverted grid test deficit of TBK1^E696K/E696K^ knock-in mice that did not reach statistical significance (Fig. S3 H). Thus, a sex-dependent effect cannot be excluded at this point. To detect FTD-like symptoms, we used a test battery consisting of the three-chamber social test (assessing social interaction), the tube test (measuring social dominance), the Y-maze test (measuring memory function), and the open field test (assessing anxiety). 18-mo-old TBK1^E696K/E696K^ knock-in mice spent significantly less time with other mice (or more time alone in the middle chamber, respectively) in the three-chamber social interaction test (Fig. S3 I), indicating impaired social interaction (Fig. S3, J–L). All three genotypes performed similarly in the other aforesaid tests suggesting no alteration in anxiety, memory function, and social dominance (Fig. S3, M–O). Overall, the results of our behavioral analysis point to social disinterest in TBK1^E696K/E696K^ knock-in mice, one prominent symptom of the behavioral variant of FTD.

The progressive motor deficits in male mice were paralleled by a 13% and 11% loss of spinal cord motor neurons in heterozygous and homozygous TBK1^E696K^ knock-in mice, respectively, compared with wt siblings at the age of 19 mo (Fig. 3, D and E). Moreover, 19-mo-old TBK1^E696K/E696K^ mice had an age-dependent 2.4-fold increase in intrinsic foot muscle denervation, which was not observed in the proximal quadriceps muscles (Fig. 3, D, F, and G). Further, we quantified the electron microscopy pictures of axons in the ventrolateral lumbar spinal cord (LSC) (Fig. 3, H–M). We detected a slightly higher axon diameter and thinner myelin sheath (according to the G ratio) in TBK1^E696K/E696K^ mice compared with wt animals, in line with previous findings in mice with double heterozygous deficiency of TBK1/TAK1 (Xu et al., 2018). The axon number was unchanged, arguing against degeneration of the corticospinal tract.

Thus, TBK1^E696K^ knock-in mice displayed an age-dependent phenotype. Although the contribution of reduced protein expression and loss of optineurin binding to the observed phenotypes cannot be fully distinguished, the homozygous p.E696K knock-in mutation results in the complete loss of TBK1/optineurin binding. This is not the case for the heterozygous TBK1 knock-out animals, which do not show any behavioral deficits (Bruno et al., 2020), indicating that the phenotypes we observed with the homozygous p.E696K mutation are not simply due to a reduced TBK1 protein level. Hence, our findings suggest that p.E696K has a potential dominant negative effect, and both reduced protein expression and loss of optineurin binding may act together.

**Figure 4.**
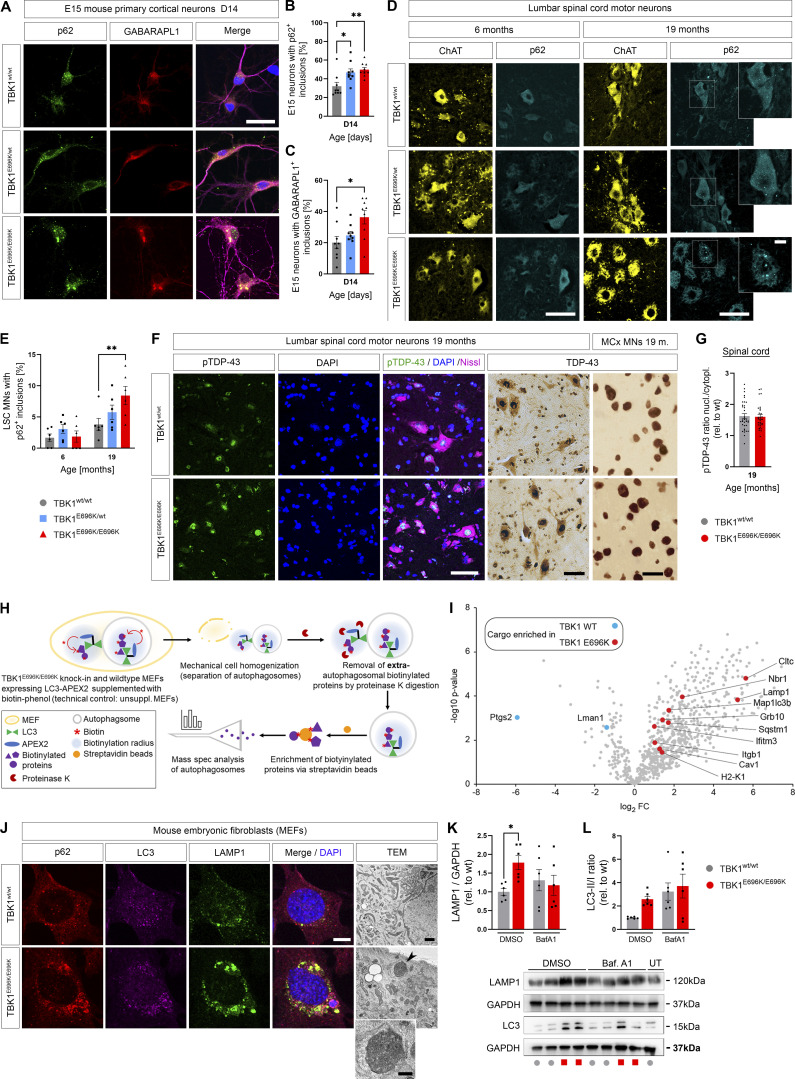
**TBK1**^**E696K/E696K**^ **knock-in impairs autophagy in cell models and mice. (A)** Representative photomicrographs of TBK1^E696K/E696K^ knock-in and wt primary cortical neurons stained against p62 and GABARAPL1. Scale bar: 10 µm. **(B and C)** Quantification of large p62^+^ and GABARAPL1^+^ inclusions in primary cortical neurons in A. Mean ± SEM of *n* = 9 embryos of mixed sex per genotype from more than three independent experiments; one-way ANOVA with post hoc Tukey’s multiple comparisons test; *P < 0.05; **P < 0.01. **(D)** Representative photomicrographs of LSC anterior horn motor neurons (MNs) of 6- and 19-mo-old TBK1^E696K^ knock-in and wt mice stained against ChAT and p62. Scale bars: 50 µm, 10 µm. **(E)** Quantification of motor neurons with large cytosolic p62 inclusions in LSC in D. Mean ± SEM of *n* = 6 male mice per genotype from two independent experiments; two-way ANOVA with post hoc Tukey’s multiple comparisons test; **P < 0.01. **(F)** Representative photomicrographs of LSC anterior horn motor neurons (MNs) and motor cortex layer five neurons 19-mo-old TBK1^E696K^ knock-in and wt mice stained against/with (p)TDP-43, Nissl, and DAPI shows no evidence of (p)TDP-43 pathology in TBK1^E696K^ knock-in mice. Scale bar: 50/50/10 µm. **(G)** The ratio of the pTDP-43 MFI nucleus/cytoplasm in LSC motor neurons is unaltered in TBK1^E696K^ knock-in mice. Mean ± SEM of pool of *n* > 29 motor neurons from *n* = 3 male mice per genotype from two independent experiments; Mann–Whitney test; ****P < 0.0001. **(H)** Scheme of protease protection coupled APEX2 proximity proteomics of autophagosomes of MEFs. **(I)** Volcano plot visualizing autophagosome content profiling in TBK1^E696K/E696K^ knock-in MEFs compared to wt. *N* = 4 technical replicates per condition from two independent experiments; multiple Student’s *t* tests with FDR correction for multiple testing; red/blue colors indicate significantly enriched proteins (P < 0.05). **(J)** Representative photomicrographs of TBK1^E696K/E696K^ knock-in and wt MEFs stained against LAMP1, LC3 and p62. Scale bar: 10 µm. Last panel: TEM of knock-in and wt MEFs shows dysmorphic and enlarged lysosomes (arrowhead and magnification) in cells with homozygous TBK1^E696K^ knock-in. Scale bar: 500 nm. **(K and L)** Western blot analysis of MEF lysates to validate the top candidates from I: LC3 and LAMP1. UT = untreated. Mean ± SEM of *n* = 6 replicates per condition from six independent experiments; two-way ANOVA with post hoc Šídák’s multiple comparisons test; *P < 0.05. Source data are available for this figure: SourceData F4.

Next, since heterozygous deletion of human *TBK1* impairs selective autophagy in iPSC-derived motor neurons in vitro (Catanese et al., 2019) and TBK1^E696K^ showed selective loss of binding to the autophagy adaptor protein optineurin (Fig. 1), we considered autophagic failure as a possible pathogenic mechanism of the p.E696K mutation. We first analyzed autophagy markers in primary cortical neurons with heterozygous and homozygous TBK1^E696K^ knock-in that showed shortened axon lengths after 14 days in culture, just like previously shown in primary neurons with heterozygous deletion of *Tbk1* (Brenner et al., 2019), supporting a neuron autonomous effect of the *TBK1* mutations (Fig. S3, P and Q). As shown in Fig. 4, A–C, primary cortical neurons prepared from mice with heterozygous or homozygous TBK1^E696K^ knock-in exhibited a higher percentage of cells with p62^+^ and GABARAPL1^+^ large inclusions than wt siblings. Staining of p62 and GABARAPL1 mostly overlapped. This finding could be translated to the in vivo situation since homozygous 19-mo-old TBK1^E696K^ knock-in mice showed a significantly higher proportion of spinal motor neurons containing cytosolic p62^+^ inclusions with a corresponding trend in heterozygous TBK1^E696K^ knock-in mice compared with wt siblings (Fig. 4, D and E). Again, these p62^+^ inclusions colocalized with GABARAPL1 staining (Fig. S3 R). Neuronal cytoplasmic inclusions or nuclear clearing of (p)TDP-43 was absent in spinal cord and motor cortex tissue of 19-mo-old TBK1^E696K^ knock-in mice (Fig. 4, F and G). This observation is in line with the vast majority of mouse models of ALS including mice with knock-in of disease-associated TARDBP mutations (Ebstein et al., 2019), and it demonstrates that neurodegeneration, neuromuscular junction (NMJ) denervation, and behavioral phenotypes can be observed in the absence of TDP-43 pathology in mice.

Since the p.E696K mutation abolishes binding of TBK1 to the autophagy adaptor protein optineurin, we hypothesized that it might impair autophagosomal targeting of specific optineurin cargos. Surprisingly, however, protease protection coupled APEX2 proximity proteomics of autophagosomes from TBK1^E696K/E696K^ and wt mouse embryonic fibroblasts (MEFs) transfected with APEX2-LC3B19 (see Materials and methods and scheme in Fig. 4 H; Zellner et al., 2021a) identified 10 proteins enriched in autophago(lyso)somes but only two with decreased abundance after false discovery rate (FDR) correction for multiple testing (Table S2). Specifically, the autophagosomal markers NBR1, LC3, clathrin, and p62, and even more the lysosomal markers LAMP1 and IFITM3 were enriched in autophagosomes from TBK1^E696K^ mutant MEFs compared with wt cells (Fig. 4 I and Table S2). Based on uncorrected P values, only six proteins tended to be decreased in autophagosomes, while 47 showed a tendency to be enriched due to the TBK1 mutation, including further autophagolysosomal markers (LAMP2, PDCD6IP, PRKAR1A, TAX1BP1, and galectin 8 [Lgals8]), the mitochondrial membrane proteins VDAC2 and VDAC3, and proteins involved in vesicle and membrane as well as cytoskeleton dynamics (Fig. S3 S and Table S2). STRING pathway analysis based on all 53 dysregulated autophagosomal proteins revealed: “protein targeting to lysosome involved in chaperone-mediated autophagy” as the top hit in the gene ontology category “biological process” (enrichment strength = 10^2,6^; FDR = 0.0041), and “lysosomal matrix” as the top hit in the category “cellular compartment” (enrichment strength = 10^2,6^; FDR = 0.00079).

Taken together, these results point to a blockade of the (autophago)lysosomal system due to TBK1^E696K^. Supporting this view, p62-, LC3-, and LAMP1-positive, large perinuclear punctae were detected in TBK1^E696K/E696K^ MEFs, indicating the accumulation of autophagosomes and lysosomes (Fig. 4 J). As shown by transmission electron microscopy (TEM), lysosomes of TBK1^E696K/E696K^ MEFs were oversized and irregularly shaped (Fig. 4 J). Consistently, the LAMP1 protein levels of TBK1^E696K/E696K^ mutant MEFs differed significantly from those of wt MEFs, but were not significantly different from those of MEFs treated with bafilomycin, which blocks the autophagosome–lysosome fusion and lysosomal acidification (Fig. 4 K). The LC3-II/I ratio showed a trend toward a difference between both genotypes under untreated conditions, but it did not reach statistical significance (Fig. 4 L).

**Figure 5.**
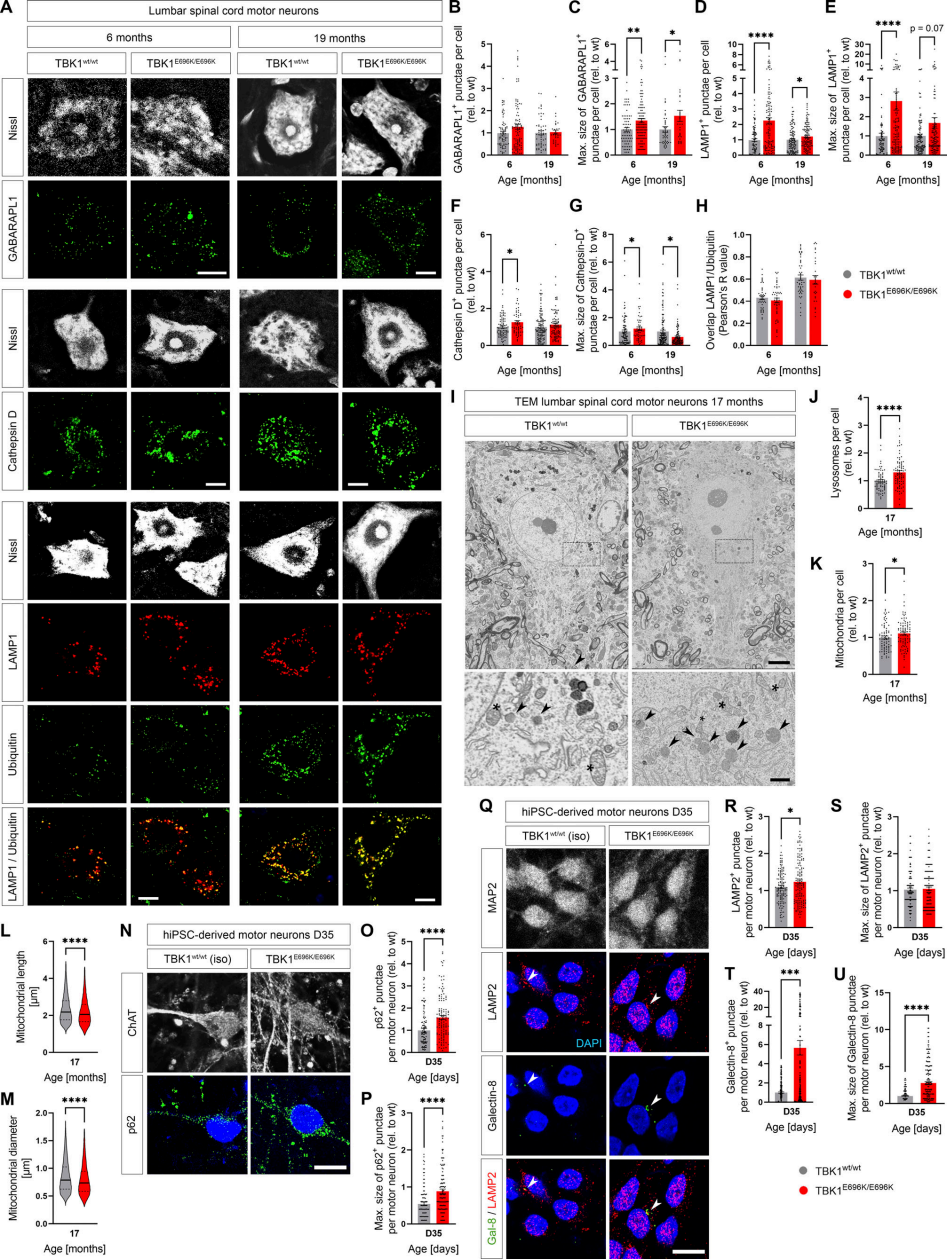
**TBK1**^**E696K/E696K**^ **knock-in causes (presymptomatic) lysosomal pathology in mice and hiPSC-derived human motor neurons. (A)** Representative photomicrographs of lumbar anterior horn motor sections from 6- and 19-mo-old homozygous TBK1^E696K^ knock-in and wt mice stained against LAMP1, GABARAPL1, cathepsin D, poly-ubiquitin, and Nissl. Scale bar: 10 µm. **(B–G)** Analysis of abundance and maximal size of GABARAPL1^+^, LAMP1^+^, and cathepsin-D^+^ punctae in lumbar spinal motor neurons in 6- and 19-mo-old TBK1^E696K/E696K^ knock-in mice compared with wt siblings shows evidence of lysosomal impairment already at the presymptomatic stage. Mean ± SEM of the pool of *n* > 30 motor neurons from *n* = 4 male mice per genotype from two independent experiments; Mann–Whitney test; *P < 0.05; **P < 0.01; ****P < 0.0001. **(H)** Analysis of overlap between LAMP1 and poly-ubiquitin shows an age- but not genotype-dependent increase in ubiquitinylation of lysosomes. Mean ± SEM of pool of *n* > 28 motor neurons from *n* = 3–4 male mice per genotype from two independent experiments; Mann–Whitney test. **(I)** Representative photomicrographs of lumbar anterior horn motor sections from 17-mo-old homozygous TBK1^E696K^ knock-in and wt mice recorded by TEM. Arrowheads indicate lysosomes; stars indicate mitochondria. Scale bars: 5 µm, 1 µm (insets). **(J and K)** The abundance of lysosomes and mitochondria in lumbar spinal motor neurons is increased in 17-mo-old TBK1^E696K/E696K^ knock-in mice; Median ± quartiles of pool of *n* > 70 motor neurons from *n* = 3 mice of mixed sex (1 female/2 males) per genotype from two independent experiments; Mann–Whitney test; *P < 0.05; ****P < 0.0001. **(L and M)** The length and diameter of mitochondria in lumbar spinal motor neurons is slightly reduced in 17-mo-old TBK1^E696K/E696K^ knock-in mice. Mean ± SEM of a pool of *n* > 13,000 mitochondria from *n* = 3 mice of mixed sex (1 female/2 males) per genotype from two independent experiments; Mann–Whitney test; ****P < 0.0001. **(N)** Representative photomicrographs of D35 hiPSC-derived motor neurons stained against ChAT and p62. Scale bar: 10 µm. **(O and P)** Analysis of abundance and maximal size of p62^+^ punctae shows an increased size and accumulation of autophagosomes in TBK1^E696K/E696K^-mutant D35 hiPSC-derived motor neurons. Mean ± SEM of pool of *n* > 90 motor neurons per genotype from three independent experiments; Mann–Whitney test; ****P < 0.0001. **(Q)** Representative photomicrographs of D35 hiPSC-derived motor neurons stained against MAP2, LAMP2, and galectin 8. Arrowheads indicate galectin 8^+^ lysosomes. Scale bar: 10 µm. **(R and S)** Analysis of abundance and maximal size of LAMP2^+^ punctae shows an accumulation of lysosomes in TBK1^E696K/E696K^-mutant D35 hiPSC-derived motor neurons. Mean ± SEM of pool of *n* > 120 motor neurons per genotype from three independent experiments; Mann–Whitney test; *P < 0.05. **(T and U)** Analysis of abundance and maximal size of galectin 8^+^ punctae shows an accumulation and increase of galectin 8^+^ organelles in TBK1^E696K/E696K^-mutant D35 hiPSC-derived motor neurons mostly colocalizing with LAMP2^+^ lysosomes. Mean ± SEM of a pool of *n* > 160 motor neurons per genotype from three independent experiments; Mann–Whitney test; ****P < 0.0001.

Similarly, cell-wise profiling of spinal cord motor neurons from 6- and 19-mo-old-mice revealed that compared with wt siblings, homozygous TBK1^E696K^ significantly increases the number and maximal size of p62^+^, GABARAPL1^+^, and LAMP1^+^ punctae (Fig. 5, A–E; and Fig. S3, T and U). For GABARAPL1 and LAMP1, this was evident already at the presymptomatic stage at 6 mo of age, supporting a causative role of early autophagolysosomal pathology for TBK1-ALS/FTD. The increased number and maximal size of LAMP1^+^ punctae per motor neuron in comparison to the respective controls appears even more pronounced at 6 mo than at 19 mo. A possible explanation for this observation could be that motor neurons with early, strong lysosomal pathology are more vulnerable and therefore less represented at 19 mo. Alternatively, increased background autofluorescence due to enhanced lipofuscin deposition could partially mask genotype-dependent effects at an advanced age. The increased abundance of lysosomes in homozygous TBK1^E696K^ knock-in mice was confirmed by TEM (Fig. 5, I and J). Importantly, we also corroborated the accumulation of p62^+^ autophagosomes and LAMP2^+^ lysosomes in TBK1^E696K/E696K^ mutant hiPSC-derived motor neurons when compared with isogenic controls (Fig. 5, N–S). Moreover, quantification of TEM images demonstrated a slight increase in the number of mitochondria per motor neuron (Fig. 5, I and K) and a small reduction in diameter and length of mitochondria in spinal motor neurons of TBK1^E696K/E696K^ mutant mice (Fig. 5, I, L, and M). Thus, the ALS/FTD-causing TBK1^E696K^ mutation leads to a general impairment of the autophagolysosomal progress with enlargement and accumulation of lysosomes and to a lesser extent accumulation of shortened mitochondria in motor neurons in mice.

Next, we stained spinal cord tissues of 6- and 19-mo-old mice for LAMP2 and cathepsin D to further assess whether the impact of the TBK1^E696K/E696K^ mutation on lysosome biology in vivo (number, size, and enzymatic content). At the presymptomatic stage of 6 mo, we found cathepsin D^+^ punctae slightly increased in number and size in motor neurons of TBK1^E696K/E696K^ mutant mice. However, at the symptomatic age of 19 mo, cathepsin D^+^ punctae were not increased in number anymore but showed markedly reduced maximal sizes in TBK1^E696K/E696K^ mutant mice compared with wt siblings. Thus, lysosomes containing the lysosomal degradative endoprotease displayed a drop in number (relative) and size (absolute) with increasing age. Since the number of LAMP1^+^ punctae (lysosomes) in TBK1^E696K/E696K^ knock-in mice continued to increase at 19 mo, this finding argues in favor of an impaired lyososomal turnover with the accumulation of lysosomes that lack their endoprotease cathepsin D. In line with our findings and their interpretation, a previous in vitro study found that that the TBK1 variant p.E696K causes impaired lysosomal turnover in vitro (Goodwin et al., 2017). The ubiquitination of lysosomes (overlap of LAMP1/poly-ubiquitin signals) remained unchanged between wt and TBK1^E696K/E696K^ spinal cord motor neurons, despite the appearance of a mild age-dependent increase in ubiquitination (Fig. 5 H).

Consequently, we sought to corroborate the hypothesis that impaired autophagy of damaged lysosomes (impaired lysophagy) hampers lysosomal turnover in TBK1^E696K/E696K^ knock-in mice. The danger receptor galectin 8 (Lgals8) targets membranes of damaged organelles including lysosomes to mediate their degradation by recruiting the autophagy receptors NDP52 and TAX1BP1 (Thurston et al., 2012; Bell et al., 2021). As shown above, both galectin 8 and TAX1BP1 accumulated in autophagosomes of TBK1^E696K/E696K^ mutant MEFs (Fig. S3 R and Table S2). Although these findings could not be analyzed in vivo (due to limited signal quality from the respective antibodies in the mouse spinal cord tissue), a highly significant accumulation of galectin 8^+^ punctae was observed in human TBK1^E696K/E696K^ mutant iPSC-derived motor neurons that largely overlapped with LAMP1 (Fig. 5, Q, T, and U). Our findings indicate that homozygosity of TBK1^E696K^ leads to impaired lysosomal degradation and accumulation of galectin 8^+^ lysosomes.

Genetic knock-out of beta-IFN signaling (which is also regulated by TBK1) has been shown to cause defects in neuronal autophagy (Ejlerskov et al., 2015). Since protein expression and thus kinase activity and IFN transcription is reduced in TBK1^E696K/E696K^ knock-in mice, this effect could in principle add to autophagy impairment caused by the disrupted interaction of TBK1^E696K^ with optineurin.

In conclusion, although our results support the known biological role of TBK1 in the autophagy pathway (Wild et al., 2011; Heo et al., 2015), the organelle-specific proteomics analysis provided new evidence for a defect of (autophago)lysosomal turnover in vitro and in vivo beyond the autophagosomal accumulation of one or few TBK1 substrates. This proposed model is in line with the observation that optineurin not only is an autophagy adaptor protein but also serves as an upstream regulator of TBK1 by recruiting the kinase to microdomains, which leads to local autophosphorylation and thereby (auto)activation of TBK1 (Heo et al., 2015; Yamano et al., 2023, *Preprint*). The early autophagosomal enrichment specifically of lysosomal proteins in the mouse spinal cord was surprising; however, it supported the recently suggested biological role of TBK1 for lysosomal acidification and lysophagy in vitro (Bussi et al., 2018; Eapen et al., 2021; Hao et al., 2021, *Preprint*). These previous reports were indeed exclusively based on in vitro neuronal or immortalized cell models and on the full deletion of *TBK1*, limiting the interpretation of the respective results in the context of ALS/FTD pathogenesis (Bussi et al., 2018; Eapen et al., 2021; Hao et al., 2021, *Preprint*). In contrast, we here provide both in vitro data based on a specific ALS/FTD *TBK1* missense mutation and respective results from a newly developed in vivo mouse model. In addition, we provide evidence for lysosomal pathology already at the presymptomatic stage in vivo in the mouse spinal cord. The observed early (autophago)lysosomal defects combined with the absence of autoimmunity and RIPK-dependent necroptosis in an in vivo model supports the conclusion that autophagy or lysophagy—rather than immune-linked functions of TBK1—are most relevant for ALS/FTD causation. Beyond that, several other ALS disease genes are functionally located in autophagy pathways (e.g., *OPTN*, *C9orf72*, *SQSTM1*, *VCP*) (Weishaupt et al., 2016), extending the significance of findings related to TBK1^E696K^ knock-in mice to a more general perspective of ALS pathogenesis.

The generation of animal models representative of the human disease is a long-standing priority of the scientific ALS community. In this context, the model described here represents a slower but molecularly probably more representative paradigm (slow but steady accumulation of pathology over a long time) that for example could turn out to have a predictive value for in vivo drug testing higher than mice with a strong overexpression of mutant SOD1 (Gurney et al., 1994). Moreover, massive glial neuroinflammation is usually not observed in human post-mortem samples. Homozygous knock-in of TBK1^E696K^ precipitates age-dependent motor neuron degeneration paralleled by progressive motor deficits, including a distal rather than proximal pattern of muscular denervation, reminiscent of the typical ALS manifestation in humans. The observations in our TBK1^E696K^ knock-in mice are thus in line with the age-dependent, milder, and probably more authentic motor phenotypes of few other ALS mouse models based on endogenous expression levels of the ALS-associated mutant proteins, such as FUS, TDP-43, and even mutant SOD1 (Fus^ΔNLS/+^ mice [Scekic-Zahirovic et al., 2016, 2017]; TDP-43^Q331K^, TDP-43^M337V^, TDP-43^G298S^ mice [Arnold et al., 2013; Ebstein et al., 2019]; SOD1^G85R^ [Dominov et al., 2023, *Preprint*]).

In summary, we generated the first mouse strain with a TBK1^E696K^ knock-in to determine the ALS/FTD-relevant functional deficits of TBK1 mutations. We show that knock-in of the TBK1^E696K^ variant, which selectively abrogates the interaction with the autophagy adaptor protein optineurin and thus leads to a partial and specific loss-of-function, is sufficient to cause a progressive, age-dependent motor neuron disease phenotype in mice. TBK1^E696K^ knock-in mice are viable and devoid of RIPK1/TNF-α dependent liver necroptosis or overt autoinflammation, contrary to mice with bi-allelic full deletion of *Tbk1* or heterozygous *Tbk1/Tak1* double mutations. In contrast to the broad biological consequences of a pleiotropic *Tbk1* full knock-out, results based on the ALS/FTD-linked point mutation p.E696K used here suggest that a limited, more specific defect of TBK1 function is responsible for causing ALS/FTD. Our in vitro and in vivo data point to an early motor neuron-autonomous (autophago)lysosomal dysfunction as a promising therapeutic target in *TBK1*-linked ALS/FTD patients.

## Materials and methods

### Characterization of TBK1 interactions with LuTHy

LuTHy-BRET and donor saturation assays were performed as described previously (Trepte et al., 2018). In brief, open reading frames of optineurin, TANK, TBK1, or the TBK1 mutants were cloned into LuTHy expression vectors (#113446, #113447, #113448, #113449; Addgene) by standard linear recombination reactions using the Gateway Cloning System (Invitrogen) and validated by restriction enzyme digest, agarose gel electrophoresis, and Sanger sequencing. Optineurin and TANK were used as donors (N- or C-terminally tagged with NanoLuc luciferase [NL]), TBK1, and TBK1 mutants as acceptors (C-terminally tagged with mCitrine-Protein A [mCit-PA]) constructs. LuTHy control vectors expressing only NL (#113442; Addgene) or PA-mCit ( #113443; Addgene) were used for the calculation of corrected scores, the PA-mCit-NL tandem construct (#113444; Addgene) as positive, and NL cotransfected with PA-mCit-only as negative controls. HEK293 cells were reverse-transfected using linear polyethyleneimine (25 kDa; 23966; Polysciences), and cells were subsequently incubated for 48 h. For LuTHy-BRET donor saturation assays, increasing acceptor expression plasmids were transfected to a constant amount of donor plasmids. For single-point LuTHy-BRET assays, the acceptor was transfected in excess in comparison with the donor expression plasmid. In-cell BRET measurements were carried out in flat-bottom white 96-well plates (655983; Greiner) with four saturation series (each in duplicates) or 24 single-point protein–protein interactions (PPIs) per plate (each PPI in triplicate). Infinite microplate readers M1000 or M1000Pro (Tecan) were used for the readouts with the following settings: fluorescence of mCitrine recorded at Ex 500/Em 530 nm, and luminescence measured using blue (370–480 nm) and green (520–570 nm) bandpass filters with 1,000 ms (LuTHy-BRET). A PPI was considered positive if its corrected BRET (cBRET) ratio was ≥0.01.

### Mice

#### Generation of TBK1^E696K^ knock-in mice

To generate a conditional knock-in of the TBK1 p.E696K variant, the sequence consisting of the wt exons 20 and 21 and a poly-adenylation signal was flanked by loxP sites (exon 21 is the last exon of *Tbk1*) (Fig. 2 B). After Cre-mediated recombination, exon 19 came into the reading frame with a downstream sequence containing exon 20 with the E696K mutation and exon 21 followed by a poly-adenylation signal, hereafter referred to as TBK1^E696K-fl^. “Floxed” TBK1^E696K-fl^ mice (C57BL/6N background) were generated by Polygene, Switzerland (Project F016; full documentation is available upon request). To generate mice with a constitutive ubiquitous knock-in of TBK1^E696K^ (hereafter referred to as TBK1^E696K^ knock-in mice), conditional “floxed” TBK1^E696K-fl^ mice were mated with CMV-Cre “deleter” mice purchased from the Jackson Laboratory (B6.C-Tg(CMV-cre)1Cgn/J; C57BL/6J background; Jax strain #006054) resulting in global Cre-recombination and transmission of the TBK1^E696K^ mutation to the germline. Mice with global constitutive knock-in of the TBK1^E696K^ mutation are hereafter referred to as TBK1^E696K^ knock-in mice. Subsequently, the CMV-Cre transgene was crossed out. Heterozygous TBK1^E696K^ knock-in mice were crossed with each other. The resulting offspring (wt, heterozygous, and homozygous TBK1^E696K^ knock-in mice) was characterized in depth.

#### Genotyping

Primers used for genotyping by PCR are given in Table 1.

**Table 1.** Primers used for genotyping by PCR

Gene	Forward	Reverse	Annealing temperature
*TBK1-E696K floxed*	5′-GCC​TCC​GGC​GGC​GTC​AAG-3′	5′-AAC​CAC​GCC​TTC​CAT​CTC​C-3′	65.3°C
*TBK1-E696K*	5′-GCC​TCC​GGC​GGC​GTC​AAG-3′	5′-CAT​CAC​TAC​TCT​TCT​G-3′	65.3°C
*Cre*	5′-GCG​GTC​TGG​CAC​TAT​C-3′	5′-GTG​AAA​CAG​CTC​ACT​T-3′	51.7°C

#### Housing

Mice were maintained at 22°C with a 14/10-h light/dark cycle and had food and water ad libitum. All animal experiments were performed in accordance with the institutional guidelines of the University of Ulm and were approved by the local authority (Regierungspräsidium Tübingen, Germany; animal permission no. 1254).

#### Handling

Before start of behavioral assessment, mice were gradually accustomed to the experimenter to decrease stress and anxiety. To this end, each mouse was handled once daily for 2 min on at least 3 consecutive days until it stopped showing anxiety-associated behavior (freezing, grooming).

#### Rotarod

At accelerating Rotarod, a mouse was placed for 5 min on the Rotarod. The Rotarod started with a speed of 4 rpm and was increased to 400 rpm over a 5 min period. The running time was measured. Every mouse was tested biweekly for three consecutive rounds and the maximum value was considered.

#### Inverted grid test

With the inverted grid test, the muscular strength of the paw muscles was tested. For this test, a mouse was placed on the home cage grid. After 5 s accommodation to the setting, the grid was carefully inverted and held ∼20 cm above the cage ground. Each of these holding periods began with all four paws of the mouse grasping the grid. The hanging time was defined as the time until the mouse fell off the inverted grid measured with a stopwatch. If a mouse did not fall off the grid during 60 s, the maximum time of 60 s was scored. Each mouse was tested in 3-mo intervals for three rounds with a time lag of at least 10 min between each round.

#### Open field test

For the open field test, a mouse was placed in an open arena and the walking track length as well as the position of the mouse in the arena was recorded for 10 min with the VIEWER software from Biobserve. As a readout for anxiety, the time spent in the center was calculated. The open-field test was repeated every 4 wk.

#### Tube dominance test

The tube dominance test was performed to assess the dominance of a mouse by measuring aggression. In this test, two unfamiliar mice with different genotypes were placed on the opposite sides of an open tube. The more aggressive and therefore more dominant mouse was crowding out the less dominant one. Every mouse was tested every 6 mo for three rounds, and the number of wins was counted.

#### Y maze spontaneous alternation test

The Y maze spontaneous alternation test was performed to assess the memory function of a mouse. In this test, a mouse was placed in a Y-shaped arena, with an angle between every arm of 120°. The pattern of arm visits was recorded for 5 min with the VIEWER software of Biobserve. Under normal circumstances, a mouse would spontaneously alternate between the arms, and if the memory function was impaired, the mouse would not remember which arm it had visited before and the probability of alternating or same-arm visits would increase. The Y maze spontaneous alternation test was repeated every 4 wk.

#### Three-chamber social test

For the three-chamber test, a mouse was placed in an arena with three chambers, where the middle chamber had closable openings to the other two chambers. In the first part of the test, the mouse could choose to be in the middle chamber or to enter another chamber where an unfamiliar mouse was placed in a little cage. In the second part of the test, the mouse had the option to choose between the middle chamber, the chamber with the mouse of part one, or the third chamber with another unfamiliar mouse. For both parts, the time the mouse spent in each chamber was recorded for 10 min with the VIEWER software from Biobserve. The three-chamber test was repeated every 3 mo.

### Tissue preparation

At the indicated time points, mice were deeply anesthetized by i.p. injection of a ketamine/rompun mixture and were transcardially perfused with 20 ml PBS and 20 ml of 4% paraformaldehyde (PFA) for fixation. Spinal cords and muscles were fixed overnight with 4% PFA, then dehydrated in 30% sucrose (Sigma-Aldrich) in PBS for 48 h at 4°C, embedded in Tissue-Tek O.C.T. compound (Sakura), and stored at −80°C until use. Embedded spinal cords were sectioned into 12-µm coronal slices using a cryotome (Leica). Serial sections covering the whole LSC were obtained from each animal. Every 10th section was chosen for quantification of anterior horn motor neurons (total of eight sections). Every 20th section was used for the quantification of microglia and astrocytes (total of four sections each). Quadriceps and foot muscles were sectioned into 25-µm longitudinal slices. At least 300 NMJs were recorded per genotype. Mice whose tissue was used for protein analysis were deeply anesthetized by i.p. injection of a ketamine/rompun mixture and were transcardially perfused with 20 ml PBS. The extracted tissue was immediately transferred to liquid nitrogen and stored at −80°C until use.

### Immunohistochemistry and immunofluorescence

Transverse sections of the spinal cord (12 µm thick) and the muscles (25 µm thick), as well as coronal sections of the brain (40-µm thick) were cut using a cryotome.

PFA-fixed cells, spinal cord, and muscle sections were blocked for 1 h using a permeabilization/blocking solution containing Tris-buffered saline (TBS) with 5% FCS and 0.25% Triton X-100 (Sigma-Aldrich). After washing once with TBS, cells/sections were stained with combinations of goat anti-ChAT (1:100; Millipore), NeuroTrace 640/660 Deep-Red Fluorescent Nissl Stain (1:100; Invitrogen), mouse anti-p62 (1:500; Abcam), rabbit anti-p62 (1:2,000; MBL), rabbit anti-GABARAPL1 (1:1,000; Proteintech), rat anti-LAMP1 (1:1,000; LAB 1D4B-C; RRID:AB_21345000 LAMP1, Hybridoma Bank, DSHB), rabbit anti-IBA1 (1:500; Wako), goat anti-IBA1 (1:1,000; Abcam), rabbit anti-PU.1 (1:100; Cell Signaling), rabbit anti-GFAP (1:750; Abcam), chicken anti-GFAP (1:1,000; Abcam), goat anti-hSOX9 (1:100; R&D Systems), α-bungarotoxin 488 (1:1,000; Invitrogen), anti-Synaptophysin (1:1,000; Abcam), rabbit anti-cathepsin D (1:100; Abcam), rabbit anti-TBK1 (1:1,500; Abcam), rabbit anti-TNF (1:100; Abcam), rabbit anti-Gal-8 (ab109519; 1:100; Abcam), rabbit anti-pTDP-43 (1:500; Proteintech), mouse anti-polyubiquitin (1:500; Enzo), rat anti-Clec7a (1:30; InvivoGen), and rabbit anti-ChAT (1:500; custom-made to EPR13024(B), kind gift by A. Catanese). Antibodies were diluted in TBS containing 0.25% Triton X-100 and 5% horse serum. Sections were incubated with the primary antibody for 12–72 h at 4°C, washed three times with TBS, and incubated with the secondary antibodies in TBS containing 0.25% Triton X-100 and 5% horse serum for 1 h at room temperature while being protected from light. Secondary antibodies used for immunofluorescence were donkey anti-rat/rabbit/mouse Alexa Fluor 488/546/647 (1:750; Invitrogen). Sections were then washed three times with TBS and coverslipped in Fluoromount G (Southern Biotech).

Spinal cord and coronal sections of the motor cortex were pretreated at 80°C in citrate buffer (pH 6), immunostained overnight at 4°C with the primary rabbit anti-IBA1 (1:1,500; WAKO), mouse anti-GFAP (1:1,000; Merck Millipore [Sigma-Aldrich]), p-RIPK1(S166) (1:100; Cell Signaling), and rabbit anti-TDP-43 (1:1,000; Proteintech) antibodies followed by incubation with a biotin-conjugated secondary antibody (anti-rabbit 1:200; Vector Laboratories). The immune reaction was visualized with an avidin–biotin–peroxidase complex (ABC Vectastain, Vector Laboratories) and the chromogen 3,3′-diaminobenzidine tetrahydrochloride (DAB; Sigma-Aldrich).

### Hematoxylin and eosin (HE) staining

In HE staining, nuclei were stained blue and the extracellular matrix and cytoplasm pink. Liver and skin samples were sliced into 12-μm sections and muscle samples into 25-μm sections using a cryotome. All tissue sections were briefly washed with distilled H_2_O to remove the Tissue-Tek O.C.T. Compound. Thereafter, the slides were incubated in Hämalaun Mayer solution for 1 min at room temperature followed by a brief dip in distilled H_2_O and two to three dips in 1% hydrochloric acid (HCl) in 70% ethanol. After a washing step for 10 min under running tap water, tissue sections were incubated with eosin for 2.5 min at room temperature. After another washing step for 10 min under running tap water, tissue sections were dehydrated in an ascending series of ethanol (50% ethanol for 1 min, 70% ethanol for 2 min, 95% ethanol for 2 min, and 100% ethanol for 2 min). Finally, the tissue sections were incubated twice for 5 min in xylene and mounted with EUKITT.

### Image and data analysis

Microphotographs of DAB-immunostained sections of the motor cortex were recorded with an upright bright field Eclipse LV100ND microscope (Nikon) or an inverted DMi8 microscope (Leica). Immunofluorescent muscle and spinal cord sections were recorded with an Axio Observer.A1 microscope (Zeiss) or a TCS SP8 confocal laser scanning microscope (Leica) using the same acquisition settings for every section. For stereological analysis, investigators were blinded to the genetic background of the animals. Nissl^+^/ChAT^+^ motor neurons, IBA1^+^/PU.1^+^ microglial cells, and GFAP^+^/SOX9^+^ astrocytic cells were counted manually with the ImageJ Cell Counter Plugin (National Institutes of Health). For the analysis of the proportion of primary cortical neurons and spinal motor neurons with p62-positive inclusions or GABARAPL1 positive inclusions, cells with p62 or GABARAPL1-positive inclusions were counted and divided by the total amount of primary cortical neurons or Nissl^+^/ChAT^+^ motor neurons respectively. For analysis of p62^+^/GABARAPL1^+^/LAMP1^+^/cathepsin D^+^/galectin 8^+^ punctae and somals sizes of microglia and astrocytes, the p62^+^/GABARAPL1^+^/LAMP1^+^/cathepsin D^+^/galectin 8^+^ area per ChAT^+^ motor neuron or the IBA1^+^ or GFAP^+^ area, respectively, was measured using the ImageJ “Threshold Color Plugin,” the “ROI Manager,” and “Particle analysis” functions. For analysis of NMJs, 25 µm z-stacks (3-µm step size) of the intrinsic foot muscles were recorded. The innervation of NMJs was assessed manually by a blinded investigator by comparing the overlap of the presynapse (neurofilaments, synaptophysin) and the postsynapse (α-bungarotoxin; fully innervated, partially innervated, fully denervated) using the ImageJ “Maximum projection” function. NMJs were assessed as “denervated” when presynaptic synaptophysin/neurofilament staining was completely absent.

Electron microscopy images were acquired with a JEOL JEM 1400plus TEM equipped with a Ruby 8 megapixel CCD camera. For quantification of the number of lysosomes and mitochondria, the functions “ROI Manager” and “Cell counter” were used. For quantitative morphological analysis of axons and mitochondria, the programs/plugins SimpliPyTEM (Ing et al., 2023, *Preprint*) and QuPath (Schmidt et al., 2018; Weigert et al., 2020) were used.

### Primary cortical neuron culture

Primary cortical neurons were dissected from E15.5 embryos. A pregnant mouse was sacrificed by cervical dislocation and the amnions including the embryos were washed in PBS. The embryos were sacrificed by decapitation. Tail biopsies were taken for genotyping. The two brain hemispheres were separated. The bulbi olfactorii and the meninges were removed. Finally, the cortex was separated from the remaining brain and stored in ice-cold PBS. To generate a cell suspension, PBS was removed, and the cortices were resuspended in 1 ml of DMEM supplemented with 10% FCS. The cell suspension was filtered through a 40-µm Flowmi cell strainer. Cells were counted and 500,000 or 125,000 cells were seeded in each well of a 12- or 24-well plate respectively. Cells were cultured in a neurobasal medium with 2% B-27 Supplement, 1% of Gluta-MAX Supplement, and 1% of penicillin-streptomycin. 50% of the medium was exchanged twice a week. Neurons were harvested for western blotting and quantitative PCR (qPCR) or stained in the14th day in vitro.

### Primary microglia culture

Primary cells were prepared from heterozygous and homozygous P0–5 pups. Tail biopsies were taken for genotyping. Microglia were prepared as previously described (Wiesner et al., 2013). Substances and solutions were from Gibco or Sigma-Aldrich. In brief, for microglia, forebrains were digested and dissociated. Cells were seeded in T25 cell culture flasks in supplemented DMEM (Gibco). Microglia were located on top of a confluent astrocyte layer. The loosely attached microglia cells started to detach and float in the culture media. At this point, microglia cells were harvested. Microglia were shaken off from the astrocyte layer by clashing the flasks and seeded on 6-well plates with 5 × 10^6^ cells. Microglia were stimulated with LPS 50 ng/ml for 6 h.

### MEF culture

MEFs were dissected from E12.5 mouse embryos. Embryos were decapitated, and their tails were taken for genotyping. The bodies of the embryos were taken, and the organs were removed. The remaining tissue was stored in ice-cold PBS. The tissue was homogenized and DMEM with 10% FCS, 1% of penicillin-streptomycin (5,000 µg/ml) was added, and the suspension was centrifuged for 10 min at 250 relative centrifugal force. Thereafter, the cells were resuspended in DMEM supplemented with 10% FCS and seeded in T25 flasks. MEFs were maintained in a DMEM medium supplemented with 10% FCS and 1% of penicillin-streptomycin. After immortalization, MEFs were passaged twice a week as soon as they were confluent.

### hiPSC-derived motor neurons

E696K-mutant hiPSCs were purchased from Jackson Laboratories. Using CRISPR/Cas technologies, Jackson Laboratories engineered cell lines with the homozygous TBK1-E696K missense mutation (IPSC,JIPSC1122_TBK1_E696K_SNV/SNV_human iPSC) and its isogenic control (IPSC,JIPSC1126_TBK1_E696K_REV/WT_human iPSC). Each cell line was differentiated into motor neurons and each experiment included three technical replicates per cell line. Motor neuron differentiation was based on the protocol from Du et al. (2015) with minor modifications, as described in Pant et al. (2022). The hiPSCs were cultured in in-house-made Essential 8 media until they reached 50% confluency. At this point, they were switched to motor neuron basal media comprising of the following components: Neurobasal medium and DMEM F12 medium without HEPES in a 1:1 ratio, 1% penicillin-streptomycin (Thermo Fisher Scientific), 0.5 N2 supplement (17502048; Thermo Fisher Scientific), 0.1 mM ascorbic acid (Sigma-Aldrich), 1% GlutaMAX (Thermo Fisher Scientific), and 0.5 × B27-Supplement (Thermo Fisher Scientific). This basal media was further supplemented with 3 μM CHIR99021 (Cell Guidance), 2 μM DMH1 (Selleck Chemicals), and 2 μM SB431542 (Cell Guidance Systems). The medium was changed daily, and the volume increased with an increase in cell density. After 6 days, cells were passaged with Accutase (Thermo Fisher Scientific). They were plated onto Geltrex-coated 6-well plates in a 1:6 ratio. The cells were cultured in basal media supplemented with 1 μM CHIR99021, 2 μM DMH1, 2 μM SB431542, 0.5 μM purmorphamine (Cell Guidance Systems), and 0.1 μM retinoic acid (Sigma-Aldrich). Media was changed daily, and the volume was increased with an increase in cell density. At the end of the 12-day induction period, cells differentiated into motor neuron precursor cells (MNPs). The MNPs were passaged using Accustase and plated onto Geltrex-coated 6-well plates in a 1:3 ratio. They were cultured in basal media supplemented with 0.1 μM purmorphamine and 0.5 μM retinoic acid. Media was changed daily, and the volume increased with an increase in cell density. After 6 days, cells were passaged using Accutase and were plated onto PEI (Sigma-Aldrich)/Laminin (Sigma-Aldrich)-coated plates. The cells were now cultured in basal media supplemented with 0.1 μM purmorphamine, 0.5 μM retinoic acid, 5 μM DAPT (Cell Guidance Systems), 1 μM LM22A (Sigma-Aldrich), 1 μM LM22B (Tocris), 10 ng/ml GDNF (Cell Guidance Systems), and 10 ng/ml IGF-1 (Cell Guidance Systems). Half the media was changed twice a week. All through the protocol, 10 μM ROCK inhibitor (Y-27632) was added to the medium for 24 h following each passaging step. Media containing light-sensitive retinoic acid was stored in vials covered with foil to protect from direct light. The MNPs were cryopreserved in basal media supplemented with 10% DMSO (Carl Roth) and 10 μM ROCK inhibitor (Y-27632) and were later used for differentiation into neurons. MNPs were also expanded and then cryopreserved for future use. They were passaged with Accutase in a 1:6 ratio and cultured in basal media supplemented with 1 μM CHIR99021, 2 μM DMH1, 2 μM SB431542, 0.5 μM purmorphamine (Cell Guidance Systems), 1 μM retinoic acid (Sigma-Aldrich), and 0.5 mM valproic acid (Thermo Fisher Scientific). They were cultured in this medium for 6 days and then cryopreserved. Differentiated motor neurons were characterized by immunostaining for choline acetyltransferase (ChAT).

### CHX treatment

To measure protein stability, cells were treated 24 h after transfection with a total concentration of 30 μg/ml of CHX. CHX was stored in stocks of 10 mg/ml in DMSO. After an incubation of 0, 3, 6, and 9 h at 37°C, cells were harvested in urea buffer.

### Western blotting

To extract protein from tissue/cells, 200 µl urea lysis buffer (8 M urea, 10 mM Tris, and 50, pH 8.0) was added to the sample and tissue was lysed using the TissueLyser II. To extract protein from cells, 100 µl urea buffer was added to the cells, and the cells were scraped off the cell culture dishes. HEPES lysis buffer (50 mM HEPES, 150 mM NaCl, 20 mM NaF, 1.5 mM MgCl_2_, 1% NP-40, 0.5% deoxycholate, 10% glycerol, 1 mM EDTA, 1 mM PMSF, 1 U benzonase and protease inhibitor cocktail [Roche], pH 7.4) was used to extract protein from HEK293 cells. Immunoblotting was performed according to standard procedures, using a total protein amount of 20–30 µg per sample and the XCell II Blot Module system (Thermo Fisher Scientific). The following antibodies were used: rabbit anti-GAPDH (1:10,000; Proteintech), mouse anti-GAPDH (1:80,000; Proteintech), rabbit anti-GFP (1:2,500; Abcam), rabbit anti-LC3B (1:1,000; Cell Signaling), rabbit anti-LC3B (1:1,000; MBL), rat anti-LAMP1 (1:2,000; DSHB), mouse anti-myc (1:2,000; Cell Signaling Technology), rabbit anti-MLKL (1:500; Proteintech), rabbit anti-RIPK1 (1:500; Cell Signaling Technology), anti-phospho-RIPK1(S166) (1:1,000; Cell Signaling), rabbit anti-TBK1 (1:1,000; Thermo Fisher Scientific), mouse anti-tubulin (1:80,000; Sigma-Aldrich), rabbit anti-TAK1 (Cell Signaling), rabbit anti-OPTN (1:2,000; Invitrogen), rabbit anti-pOPTN(Ser177) (1:1,000; Cell Signaling), goat anti-mouse-HRP (1:1,000; Life Technologies), and goat anti-rabbit-HRP (1:1,000, Life Technologies). Super Signal West Pico Chemiluminescent Substrate (Thermo Fisher Scientific) or WesternBright Chemiluminescent Substrate (Biozym) were used for enhanced chemiluminescent detection in the FUSION SOLO S (Peqlab) or the LAS-3000 (Fujifilm) systems.

### Real-time PCR

Total RNA was isolated from primary cultures or mouse tissue using the QIAGEN RNeasy Plus Mini Kit for cells and the RNeasy Lipid Tissue Mini Kit for tissue samples. Reverse transcription reactions were performed with the QuantiTect Reverse Transcription Kit (#205311; Qiagen) according to the manufacturer’s instructions. Subsequent PCR reactions were performed in duplicates on a CFX96 real-time system (Bio-Rad) using the QuantiTect SYBR Green PCR Kit (#204143; Qiagen) and QuantiTect Primer Assays (Qiagen). The following QuantiTect primer assays were used: *Polr2a* (#QT00197757) *Ywhaz* (#QT00105350), *Tbp* (#QT00198443), *Tbk1* (#QT00121135),* Optn* (#QT00134078), *Ccl2* (#QT00167832), *Tnf* (#QT00104006), *Irf7* (#QT00245266), *Stat2* (#QT00160216), *Isg15* (#QT01772876), and *Ifnb1* (#QT00249662). The resulting Ct values were normalized to three housekeeping genes (*Polr2a*, *Ywhaz*, and *Tbp*) using the 2−ΔΔCt method (Livak and Schmittgen, 2001).

### Proteomic profiling of autophagosomes

Coupling proximity biotinylation with proteinase K digestion and mass spectrometry is a useful tool to study the content of a transient membrane compartment such as autophagosomes. Autophagosome content profiling was carried out as described before (Zellner et al., 2021b). To identify cargo in autophagosomes, we lentivirally transfected MEFs from TBK1^wt/wt^ and TBK1^E696K/E696K^ mice with a construct containing LC3B as a N-terminal fusion to the engineered peroxidase APEX2 (hereafter referred to as APEX2-LC3B) (also see scheme in Fig. 4 H). In brief, APEX2-LC3B stably expressing MEFs from TBK1^wt/wt^ and TBK1^E696K/E696K^ mice separately were supplemented with 500 µM biotin–phenol (IrisBiotech) for 30 min at 37°C before the addition of 1 mM H_2_O_2_ at room temperature. APEX2-LC3B expressing TBK1^wt/wt^ MEFs that had not been supplemented with biotin–phenol served as technical control. Cells were then first washed with quencher solution (1 mM sodium azide, 10 mM sodium ascorbate, and 5 mM Trolox in DPBS), then with DPBS, scraped, and harvested. All subsequent steps were carried out at 4°C unless stated otherwise. Cells were washed and suspended in homogenization buffer I (10 mM KCl, 1.5 mM MgCl_2_, 10 mM HEPES-KOH, and 1 mM DTT pH 7.5). After 20 min of incubation in an overhead shaker, cells were dounced with a tight-fitting pestle and mixed with homogenization buffer II (375 mM KCl, 22.5 mM MgCl_2_, 220 mM HEPES-KOH and 0.5 mM DTT pH 7.5) at a ratio 1:5 (homogenization buffer I:II). Cleared lysates were obtained by centrifugation at 600 *g* for 10 min. Samples were then treated with 100 µg/ml Proteinase K for 1 h at 37°C. Digested material was separated from membrane-protected material by centrifugation at 17,000 *g* for 15 min. Pellets were suspended in radioimmunoprecipitation assay (RIPA) buffer containing quenching components (50 mM Tris, 150 mM NaCl, 0.1% SDS, 0.5% sodium deoxycholate, 1% Triton X-100, 1× protease inhibitors [Roche], 1× PhosStop [Roche], 1 mM sodium azide, 10 mM sodium ascorbate, and 1 mM Trolox), briefly sonicated, and cleared by centrifugation at 10,000 *g*. Supernatants were then incubated overnight on pre-equilibrated streptavidin-agarose (Sigma-Aldrich). Subsequently, samples were washed three times in RIPA buffer with quenching components and three times in 3 M urea buffer (in 50 mM NH_4_HCO_3_) prior to incubation with TCEP (5 mM final) for 30 min at 55°C and shaking. Samples were alkylated with IAA (10 mM final) for 20 min at room temperature, quenched by the addition of dithiothreitol (DTT) (20 mM final) followed by two washes with 2 M urea buffer (in 50 mM NH_4_HCO_3_) and overnight trypsin digestion with 1 µg trypsin per 20 µl beads at 37°C. Supernatants were collected from the resin plus two additional washes with 2 M urea buffer, acidified with trifluoroacetic acid (1% final), and their volume decreased by vacuum centrifugation. Digested peptides were desalted on custom-made C18 stage tips and reconstituted with 0.5% acetic acid for MS analysis. Samples were loaded onto 75 µm × 15 cm fused silica capillaries (custom-made) packed with C18AQ resin (Reprosil-Pur 120, 1.9 µm, Dr. Maisch HPLC) using an Easy-nLC1200 liquid chromatography. Peptide mixtures were separated at a 400 nl/min flow rate using a 35-min acetonitrile (ACN) gradient in 0.5% acetic acid (5–38% ACN gradient for 23 min followed by 38–60% ACN gradient for 3 min and 60–95% ACN gradient for 2 min plus another 3 min at 95% CAN prior to 95–5% gradient for 2 min and another 2 min at 5% ACN) and detected on a Q Exactive HF mass spectrometer (Thermo Fisher Scientific). Dynamic exclusion was enabled for 20 s and singly charged species, charge states above eight, or species for which a charge could not be assigned were rejected. MS raw data was analyzed using MaxQuant (version 1.6.0.1) and a human Uniprot FASTA reference proteome (UP000005640) in reverted decoy mode with the following allowance: methionine oxidation and protein N-terminus acetylation as variable modifications, cysteine carbamidomethylation as fixed modifications, two missed cleavages, and five modifications per peptide, minimum peptide length of seven amino acids, first search peptide tolerance of ±20 ppm, main search peptide tolerance of ±4.5, match between runs, label-free quantification (LFQ), as well as protein, peptide and site level FDRs of 0.01. For further processing, MaxQuant output files (proteingroups) were loaded into Perseus (version 1.6.5.0) where matches to common contaminants, reverse identifications, identifications based only on site-specific modifications and with less than two peptides, and MS/MS counts were removed. Only proteins with LFQ intensities in three out of four biological replicates in at least one experimental group were kept for the subsequent LFQ. LFQ intensities were log_2_ transformed, and missing values were replaced with random numbers drawn from a normal distribution. Student’s *t* tests were used to determine the statistical significance of the abundance alterations. All proteins with P values ≤0.05 and *T* test differences ≥0.5 or ≤-0.5 in the comparison of TBK1 wt versus E696K were retained as candidates. APEX2-LC3B expressing MEFs from TBK1^wt/wt^ and TBK1^E696K/E696K^ mice supplemented with biotin were compared. APEX2-LC3B expressing TBK1^wt/wt^ MEFs that had not been supplemented with biotin–phenol served as technical control. However, only proteins with P values ≤0.05 and *T* test differences ≥0.5 or less than or equal to −0.5 in the comparison of TBK1 with biotin–phenol (i.e., the technical control) versus without biotin–phenol were classified as cargo enriched in TBK1 wt and E696K, respectively.

### Proteomics of spinal cord lysates

#### Sample extraction and lysis

Deeply anesthetized mice were transcardially perfused with PBS. CNS tissue was snap-frozen with liquid nitrogen and stored at −80°C until analysis. Samples were lysed with lysis buffer (2% SDS, 50 mM Tris-HCl pH 8.5, 10 mM TCEP, 40 mM chloroacetamide, and protease inhibitor cocktail tablet [EDTA-free, Roche]). Samples were incubated for 5 min at 95°C before sonication with Sonic Vibra Cell at 1 s ON/1 s OFF pulse for 30 s at a maximal amplitude of 30% to shear genomic DNA. After sonication, samples were incubated for 10 min at 95°C.

#### Sample preparation for liquid chromatography with tandem mass spectrometry (LC-MS/MS)

Proteins were precipitated using 3 vol of ice-cold methanol, 1 vol of chloroform, and 2.5 vol ddH_2_O. After centrifugation at 14,000 *g* for 45 min at 4°C, the upper aqueous phase was aspirated and 3 vol of ice-cold methanol were added. Samples were mixed and proteins were pelleted by centrifugation at 14,000 *g* for 5 min at 4°C. The supernatant was discarded, and pellets were washed one additional time with ice-cold methanol. Protein pellets were dried at room temperature (RT) for further use. Proteins were resuspended in 8 M urea, 50 mM Tris pH 8.2, and protein concentration determined using a BCA assay (23225; Thermo Fisher Scientific). Samples were then diluted to 4 M urea using digestion buffer (50 mM Tris pH 8.2) and incubated with LysC (Wako Chemicals) at 1:50 (wt/wt) ratio for 4 h at 37°C and were further diluted to 1 M urea using digestion buffer with 1 mM CaCl_2_ final concentration and incubated at a 1:100 (wt/wt) ratio of Trypsin (V5113; Promega) overnight at 37°C. Digests were acidified using trifluoroaceticacid (TFA) to a pH of 2–3 and peptides were purified using Empore C18 (Octadecyl) resin material (3 M Empore). The material was activated with methanol, followed by one wash each with 70% ACN/0.1% TFA and 0.1% TFA. Samples were resuspended in 0.1% TFA and loaded to resin material. Peptides were washed with 0.1% TFA and eluted with 70% ACN. Eluates were dried and stored for further processing.

Peptides were resuspended in TMT labeling buffer (0.2 M EPPS pH 8.2, 10% ACN) and peptide concentration was determined by µBCA (23235; Thermo Fisher Scientific). Peptides were mixed with TMT reagents (90111, A37724, 90061; Thermo Fisher Scientific) in 1:2 (wt/wt) ratio (2 µg TMT reagent per 1 µg peptide). Reactions were incubated for 1 h at RT and subsequently quenched by the addition of hydroxylamine to a final concentration of 0.5% at RT for 15 min. Samples were pooled in equimolar ratio (unless stated otherwise), acidified, and dried for further processing.

Before MS analysis, peptide samples were purified using Empore C18 (Octadecyl) resin material (3 M Empore) as described before, except peptides were resuspended in 3% ACN/0.1% TFA and washed with 3% ACN/0.1% TFA. After elution, samples were dried and resuspended in 2% ACN/1% formic acid (FA) for LC-MS^2/3^.

Peptides were fractionated using a Dionex Ultimate 3000 analytical HPLC. For high pH reversed-phase fractionation on the Dionex HPLC, 500 μg of pooled and purified TMT-labeled samples were resuspended in 10 mM ammonium-bicarbonate (ABC), 5% ACN, and separated on a 250 mm long C18 column (Aeris Peptide XB-C18, 4.6 mm ID, 2.6 μm particle size; Phenomenex) using a multistep gradient from 100% Solvent A (5% ACN, 10 mM ABC in water) to 60% Solvent B (90% ACN, 10 mM ABC in water) over 70 min. Eluting peptides were collected every 45 s into a total of 96 fractions, which were cross-concatenated into 12 fractions and dried for further processing.

#### Mass spectrometry

All mass spectrometry data were acquired in centroid mode on an Orbitrap Fusion Lumos mass spectrometer hyphenated to an easy-nLC 1200 nano HPLC system using a nanoFlex ion source (Thermo Fisher Scientific) applying a spray voltage of 2.6 kV with the transfer tube heated to 300°C and a funnel RF of 30%. Internal mass calibration was enabled (lock mass 445.12003 m/z). Peptides were separated on a self-made 32-cm long, 75-µm ID fused-silica column, packed in-house with 1.9 µm C18 particles (ReproSil-Pur, Dr. Maisch) and heated to 50°C using an integrated column oven (Sonation). HPLC solvents consisted of 0.1% FA in water (Buffer A), and 0.1% FA and 80% ACN in water (Buffer B).

For total proteome analysis, a synchronous precursor selection (SPS) multi-notch MS3 method was used to minimize ratio compression as previously described (McAlister et al., 2014). Individual peptide fractions were eluted by a non-linear gradient from 4 to 60% B over 217 min followed by a step-wise increase to 95% B in 7 min which was held for another 8 min. Full-scan MS spectra (350–1,400 m/z) were acquired with a resolution of 120,000 at m/z 200, maximum injection time of 50 ms and AGC target value of 4 × 10^5^. The 20 most intense precursors with a charge state between two and six per full scan were selected for fragmentation (“Top 20”) and isolated with a quadrupole isolation window of 0.4 Th. MS2 scans were performed in the Ion trap (Turbo) using a maximum injection time of 120 ms and AGC target value of 2 × 10^4^ and fragmented using CID with normalized collision energy (NCE) of 35%. SPS-MS3 scans for quantification were performed on the 10 most intense MS2 fragment ions with an isolation window of 0.7 Th (MS) and 2 m/z (MS2). Ions were fragmented using HCD with an NCE of 60% and analyzed in the Orbitrap with a resolution of 50,000 at m/z 200, a scan range of 100–1,000 m/z, AGC target value of 1.5 × 10^5^, and a maximum injection time of 150 ms. Repeated sequencing of already acquired precursors was limited by setting a dynamic exclusion of 60 s and 7 ppm and advanced peak determination was deactivated.

#### Processing of raw files

Raw files were analyzed using Proteome Discoverer (PD) 2.2 software (Thermo Fisher Scientific). Files were recalibrated using the *Homo sapiens* SwissProt database (TaxID:9606, version 2017-06-07) with methionine oxidation (+15.995) as dynamic modification and carbamidomethyl (Cys, +57.021464), TMT6 (N-terminal, +229.1629) and TMT6 (+229.1629) at lysines as fixed modifications. Spectra were selected using default settings and database searches were performed using SequestHT node in PD. Database searches were performed against trypsin digested *H. sapiens* SwissProt database and FASTA files of common contaminants (“contaminants.fasta” provided with MaxQuant) for quality control. Fixed modifications were set as TMT6 at the N terminus and carbamidomethyl at cysteine residues. As dynamic modifications TMT6 (K) and methionine oxidation were set, after search, posterior error probabilities were calculated and PSMs were filtered using Percolator using default settings. Consensus Workflow for reporter ion quantification was performed with default settings, except the minimal signal-to-noise ratio was set to 10. Results were then exported to Excel files for further processing.

The complete dataset and analysis including a graphical scheme of the analysis workflow can be found in Table S1. PCA across all proteins in all mice per genotype (*n* = 6) was performed using the ClustVis web tool (Metsalu and Vilo, 2015). We used the gene/protein list of the nCounter Mouse Glial Profiling Panel to select 757 proteins expressed in neuroglia. 360 proteins from this panel could be detected in our proteomic data. PCA and unbiased hierarchical cluster (heatmap) analysis of glial genes were performed using ClustVis web tool (Metsalu and Vilo, 2015). Statistical significant changes between samples were determined using a two-sample *t* test with a permutation based FDR of 5% on log_2_ transformed values.

### TEM

#### In vitro

Freshly plasma-coated ACLAR (plastic) films (Science Services) were put into the cell culture dish before seeding of TBK1^wt/wt^ and TBK1^E696K/E696K^ MEFs. 5% glutaraldehyde in 0.2 M cacodylate buffer prewarmed to 37°C was added 1:1 to the cell culture medium and replaced by 2.5% glutaraldehyde in 0.1 M cacodylate buffer after 5 min. Dishes were incubated for a further 25 min on ice. Cells were washed 3 × 5 min with 0.1 M cacodylate buffer on ice and stored in buffer at 4°C until post-fixation. After washes in 0.1 M sodium cacodylate buffer, post-fixation in reduced osmium (2% osmium, 2.5% potassium ferrocyanide in 0.1 M cacodylate buffer) was followed by en bloc uranyl acetate (1% aqueous uranyl acetate) contrasting, graded dehydration in ethanol, and embedding in epon resin (Serva). After ultrathin sectioning, the grids (UC7 Ultramicrotome; Leica) were contrasted by 1% uranyl acetate and lead citrate (Ultrastain; Leica). Semithin sections were contrasted by an equimolar mixture containing 1% methylene blue (Carl Roth GmbH & Co. Kg) in 100 ml sodium tetraborate and 1% (1 g) azure Blue (Carl Roth) in 100 ml water.

#### In vivo

Mouse spinal cords were fixed in 2.5% glutaraldehyde and 4% PFA in 0.1 M sodium cacodylate buffer at pH 7.4 after deep anesthesia perfusion. Spinal cords were vibratome-sectioned and immersion fixed in the same buffer for 24 h at 4°C. After tissue trimming and washes in 0.1 M sodium cacodylate buffer, postfixation in reduced osmium (2% osmium, 2.5% potassium ferrocyanide in 0.1 M cacodylate buffer) was followed by en bloc uranyl acetate (1% aqueous uranyl acetate) contrasting, graded dehydration in ethanol, and embedding in epon resin (Serva). After ultrathin sectioning, the grids (UC7 Ultramicrotome; Leica) were contrasted by 1% uranyl acetate and lead citrate (Ultrostain; Leica). Semithin sections were contrasted by an equimolar mixture containing 1% methylene blue (Carl Roth GmbH & Co. Kg) in 100 ml sodium tetraborate and 1% (1 g) azure Blue (Carl Roth) in 100 ml water.

### Statistics

For comparison of three groups and normal distribution of data, the statistical significance of endpoints was evaluated by one-way (unpaired) or two-way (paired) ANOVA followed by Tukey’s multiple comparisons post hoc test. For comparison of three groups without normal distribution of data, Kruskal–Wallis test followed by Dunn’s multiple comparisons post hoc test was used. For comparison of two groups and normal distribution of data, unpaired two-tailed Student’s *t* test or two-way ANOVA followed by Šídák's multiple comparisons test (paired analysis) was used. For comparison of two groups without a normal distribution of data, Mann–Whitney test was used. For analysis of the lifespan the log-rank (Mantel–Cox) test was used. Data are presented as median ± quartiles or means ± SEM in bar graphs. Statistical significance was reported by the P value of the statistical test procedures and was assessed as significant (*, P < 0.05), strongly significant (**, P < 0.01), or highly significant (***, P < 0.001; ****, P < 0.0001). All statistical analyses were performed with Prism software (version 9.1.0; GraphPad Software).

### Online supplemental material

Fig. S1 shows analysis of skin morphology, liver and spleen weight, and microglia count, morphology, and activation in the LSC and motor cortex (immunohistochemistry and immunofluorescence) from TBK1^E696K^ knock-in and wt siblings. Fig. S2 shows the analysis of astrocyte count and morphology in the LSC and motor cortex as well as proteomic analysis of glial markers in the LSC from TBK1^E696K^ knock-in and wt siblings. Fig. S3 provides additional data on the analysis of necroptosis, autophagy, and axon pathology as well as behavioral testing of TBK1^E696K^ mutant and wt mice, MEFs, and primary neurons, respectively. Table S1 contains the analysis of proteomics of spinal cord lysates. Table S2 contains the analysis of autophagosome-specific proteomics.

## Supplementary Material

Table S1 shows proteomics of spinal cord lysates.

Table S2 shows autophagosome-specific proteomics.

SourceData F1 contains original blots for Fig. 1.

SourceData F2 contains original blots for Fig. 2.

SourceData F4 contains original blots for Fig. 4.

SourceData FS3 contains original blots Fig. S3.

## Data Availability

The data of Fig. 4 I and Fig. S3 S are available in Table S2. The data of Fig. S2, G–I are available in Table S1. The underlying mass spectrometry proteomics raw data have been deposited to the ProteomeXchange Consortium via the PRIDE (Perez-Riverol et al., 2022) partner repository with the dataset identifier PXD048795 and PXD050731. All other data are available in the article itself, its supplementary figures, and in Zenodo (https://doi.org/10.5281/zenodo.10511307; Brenner, 2024).
