# Supplementary figures and images for: A TBK1 variant causes autophagolysosomal and motoneuron pathology without neuroinflammation in mice
Source: J Exp Med. 2024 Mar 22;221(5):e20221190. doi: 10.1084/jem.20221190 (PMC10959724; doi:10.1084/jem.20221190)

Source data for Fig. 1

Fig. 1 E

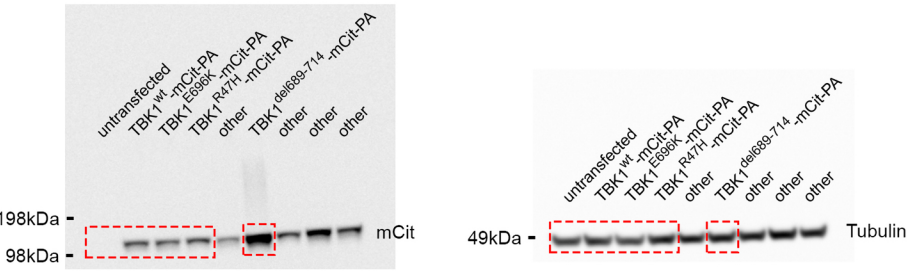

Supplement: SourceData F1 — contains original blots for Fig. 1. [file JEM_20221190_SourceDataF1.pdf]

Fig. 2 H

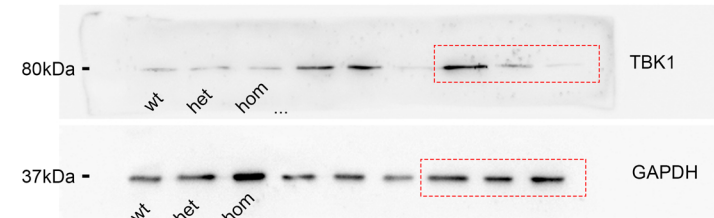

Fig. 2 I

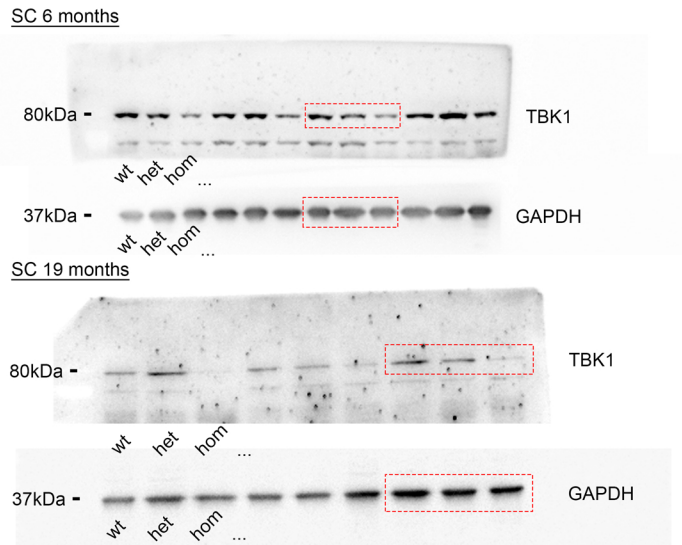

Fig. 2 P

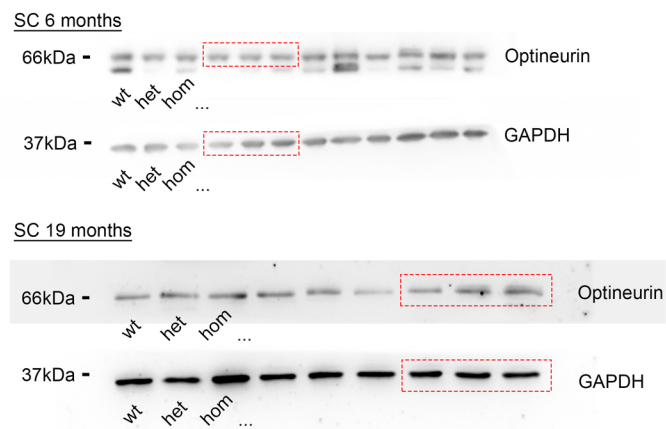

Fig. 2 R

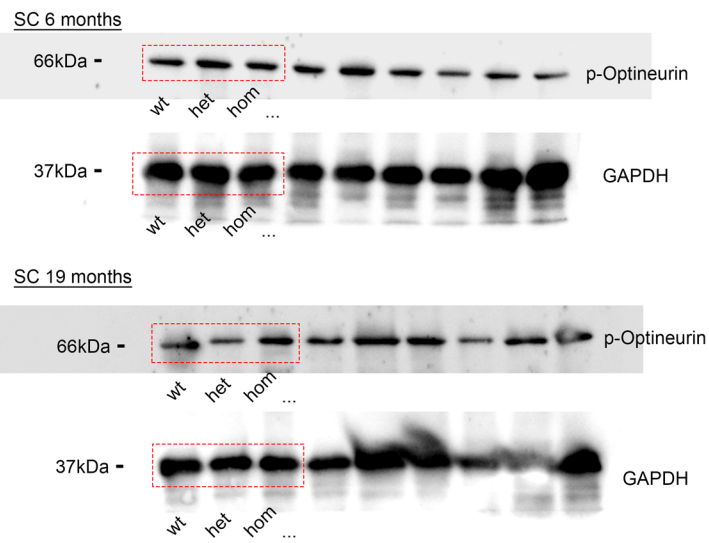

Fig. 2 O

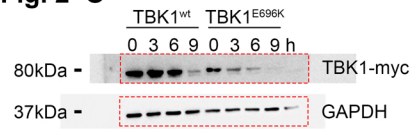

Fig. 2 J

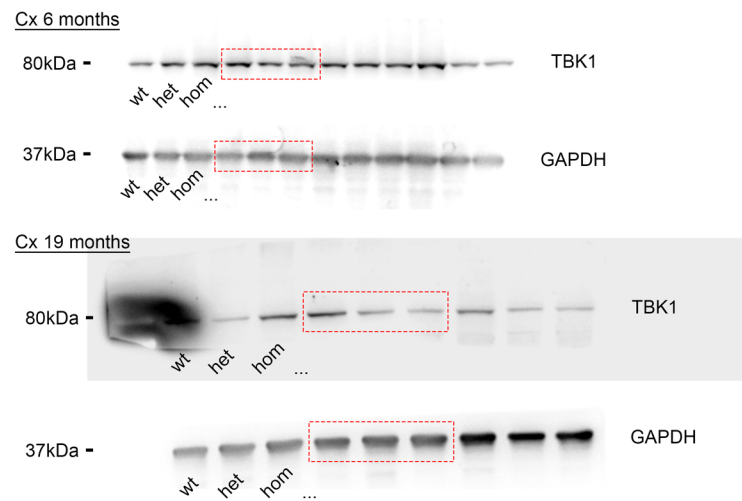

Fig. 2 Q

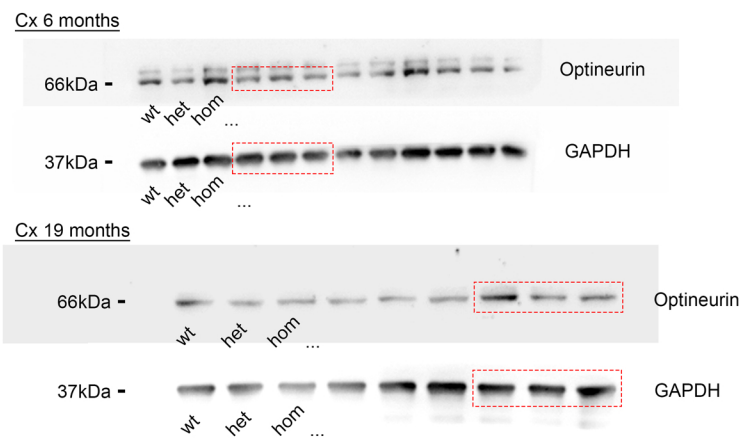

Fig. 2 S

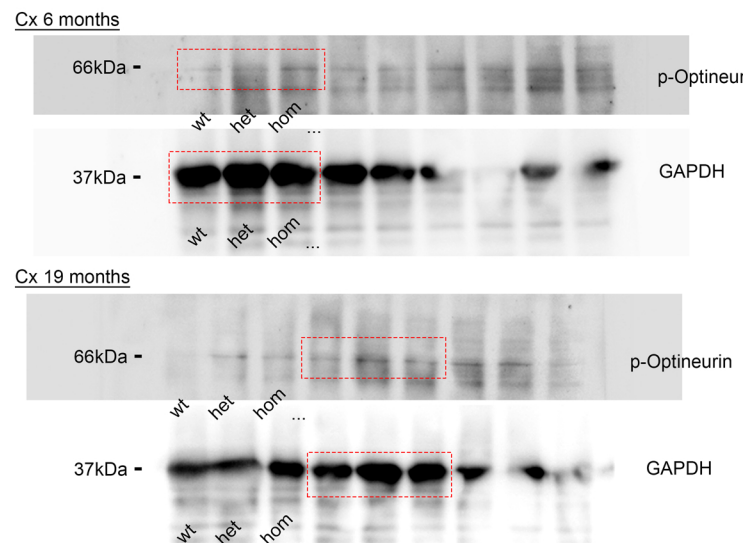

Supplement: SourceData F2 — contains original blots for Fig. 2. [file JEM_20221190_SourceDataF2.pdf]

Source data for Fig. 4

Fig. 4 K

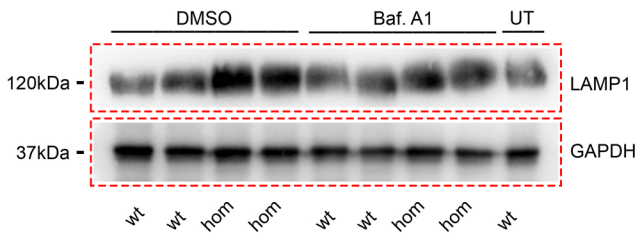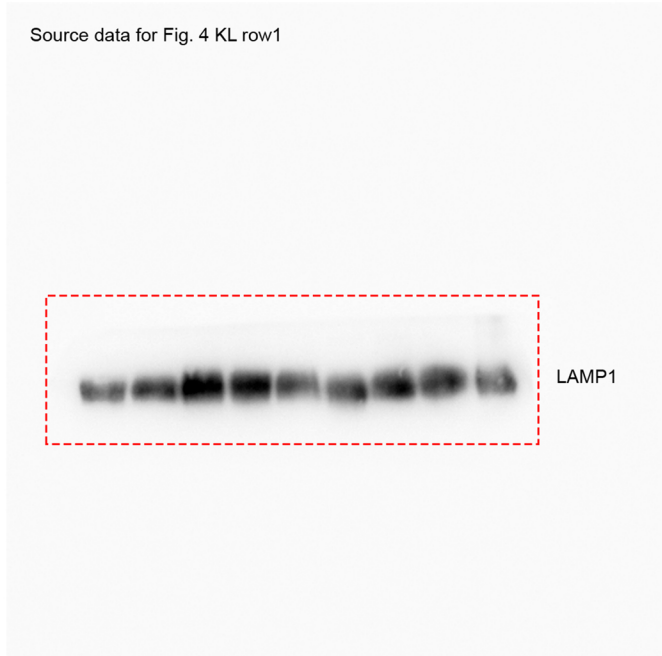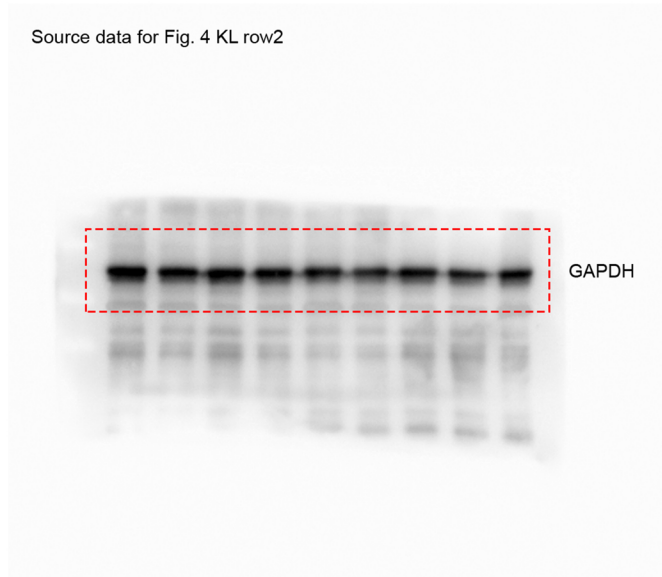

Fig. 4 L

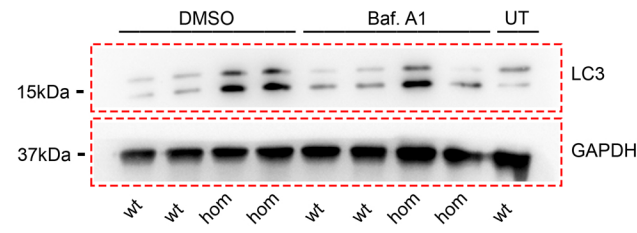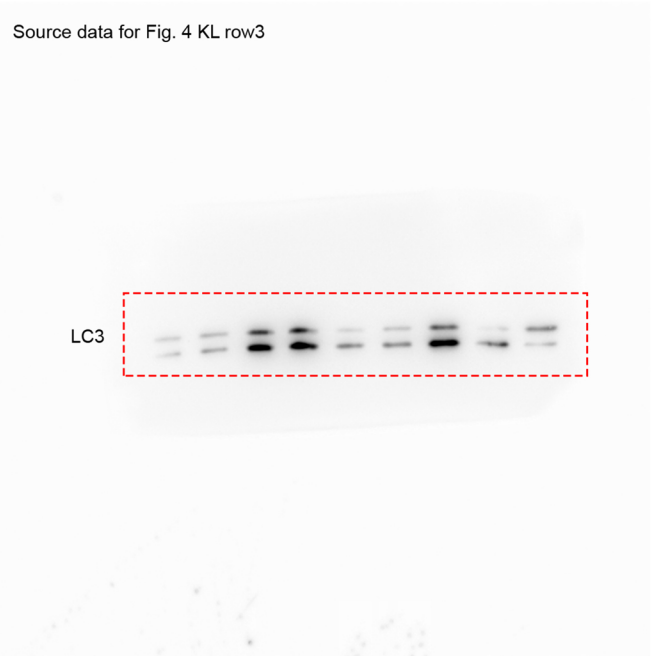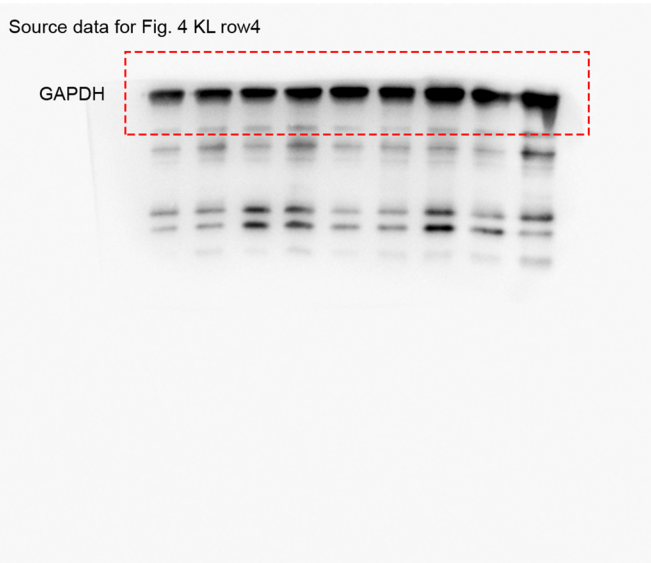

Supplement: SourceData F4 — contains original blots for Fig. 4. [file JEM_20221190_SourceDataF4.pdf]
