# Supplementary material for: A TBK1 variant causes autophagolysosomal and motoneuron pathology without neuroinflammation in mice
Source: J Exp Med. 2024 Mar 22;221(5):e20221190. doi: 10.1084/jem.20221190 (PMC10959724; doi:10.1084/jem.20221190)

Source data for Suppl. Fig. 3

Suppl. Fig. 3 A

SC 6 months

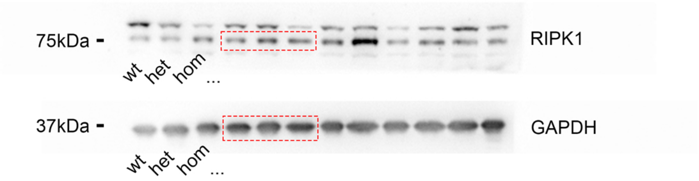

SC 19 months

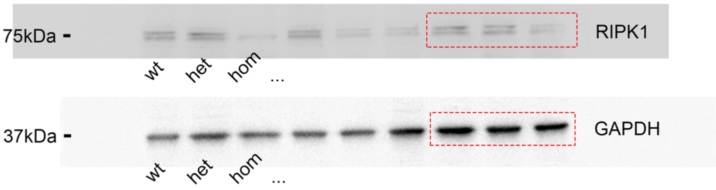

Suppl. Fig. 3 B

Cx 6 months

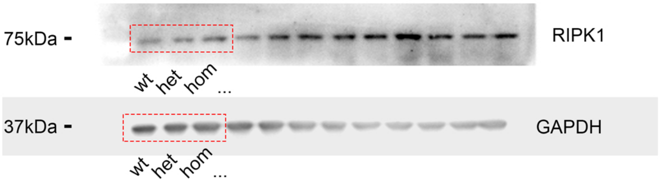

Cx 19 months

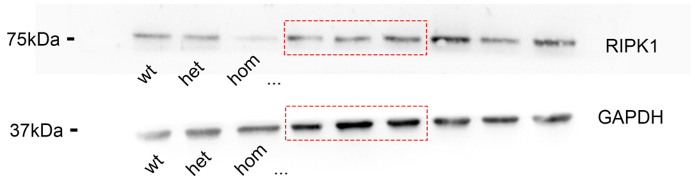

Suppl. Fig. 3 C

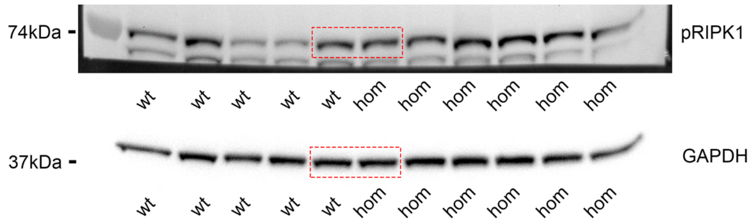

Suppl. Fig. 3 D

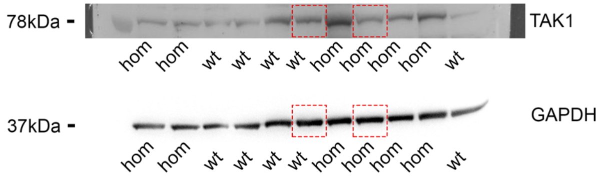

Suppl. Fig. 3 F

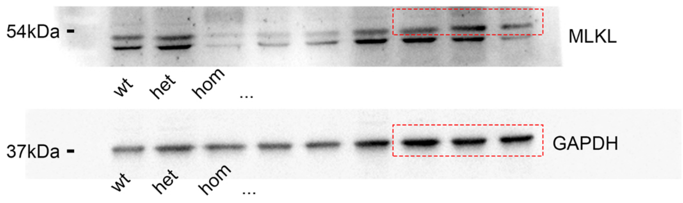

Suppl. Fig. 3 G

wt het ...

Cx 6 months

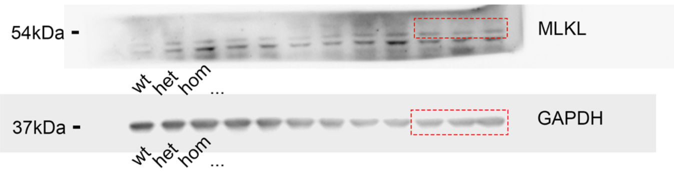

Cx 19 months

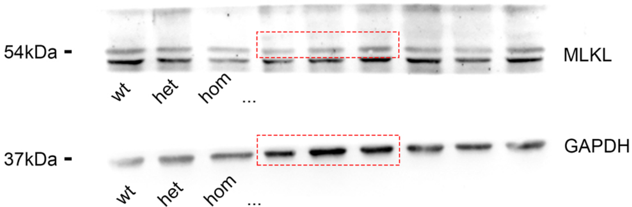

Supplement: SourceData FS3 — contains original blots Fig. S3. [file JEM_20221190_SourceDataFS3.pdf]
